# Epithelial Cells Expressing EhADH, An *Entamoeba histolytica* Adhesin, Exhibit Increased Tight Junction Proteins

**DOI:** 10.3389/fcimb.2018.00340

**Published:** 2018-09-28

**Authors:** Abigail Betanzos, Dxinegueela Zanatta, Cecilia Bañuelos, Elizabeth Hernández-Nava, Patricia Cuellar, Esther Orozco

**Affiliations:** ^1^Consejo Nacional de Ciencia y Tecnología, Mexico City, Mexico; ^2^Departamento de Infectómica y Patogénesis Molecular, Centro de Investigación y de Estudios Avanzados del Instituto Politécnico Nacional, Mexico City, Mexico; ^3^Coordinación General de Programas de Posgrado Multidisciplinarios, Programa de Doctorado Transdisciplinario en Desarrollo Científico y Tecnológico para la Sociedad, Centro de Investigación y de Estudios Avanzados del Instituto Politécnico Nacional, Mexico City, Mexico; ^4^Programa de Cáncer de Ovario, Instituto Nacional de Cancerología, Mexico City, Mexico; ^5^Centro Regional de Educación Superior, Universidad Autónoma de Guerrero, Chilpancingo, Mexico

**Keywords:** MDCK cells, EhCPADH, amoebiasis, trophozoites, epithelial barrier, transepithelial electrical resistance, intercellular junctions

## Abstract

In *Entamoeba histolytica*, the EhADH adhesin together with the EhCP112 cysteine protease, form a 124 kDa complex named EhCPADH. This complex participates in trophozoite adherence, phagocytosis and cytolysis of target cells. EhCPADH and EhCP112 are both involved on epithelium damage, by opening tight junctions (TJ) and reaching other intercellular junctions. EhADH is a scaffold protein belonging to the ALIX family that contains a Bro1 domain, expresses at plasma membrane, endosomes and cytoplasm of trophozoites, and is also secreted to the medium. Contribution of EhADH to TJ opening still remains unknown. In this paper, to elucidate the role of EhADH on epithelium injury, we followed two strategies: producing a recombinant protein (rEhADH) and transfecting the *ehadh* gene in MDCK cells. Results from the first strategy revealed that rEhADH reached the intercellular space of epithelial cells and co-localized with claudin-1 and occludin at TJ region; later, rEhADH was mainly internalized by clathrin-coated vesicles. In the second strategy, MDCK cells expressing EhADH (MDCK-EhADH) showed the adhesin at plasma membrane. In addition, MDCK-EHADH cells exhibited adhesive features, producing epithelial aggregation and adherence to erythrocytes, as described in trophozoites. Surprisingly, the adhesin expression produced an increase of claudin-1, occludin, ZO-1 and ZO-2 at TJ, and also the transepithelial electric resistance (TEER), which is a measure of TJ gate function. Moreover, MDCK-EhADH cells resulted more susceptible to trophozoites attack, as showed by TEER and cytopathic experiments. Overall, our results indicated that EhADH disturbed TJ from the extracellular space and also intracellularly, suggesting that EhADH affects by itself TJ proteins, and possibly synergizes the action of other parasite molecules during epithelial invasion.

## Introduction

*Entamoeba histolytica* is the protozoan responsible for human amoebiasis that infects 50 million people and kills between 30 and 100 thousand individuals around the world (Singh et al., 2016). Amoebiasis is characterized by acute diarrhea due to the substantial damage of the colonic epithelium produced by *E. histolytica* trophozoites (Cornick and Chadee, 2017). Trophozoites attach to and displace over the epithelium, contacting the epithelial cell surface. Then, they open the intercellular spaces by gradual separation of adjacent cells. Subsequently, epithelial cells are detached from the substrate and phagocytosed by the parasite (Martínez-Palomo et al., 1985). Several molecules are involved in this process, such as Gal/GalNAc lectin, amoebapores, cysteine and serine proteases, prostaglandin E2 (PGE2), the EhCPADH complex, among others (Chadee et al., 1987; Leippe, 1997; García-Rivera et al., 1999; Meléndez-López et al., 2007; Lejeune et al., 2011; Cornick et al., 2016).

Tight junctions (TJ) regulate ion and macromolecules flux across the epithelium, and also constitute the first barrier that pathogens face during host invasion. TJ are composed by integral proteins (e.g., claudins, occludin and junctional adhesion molecules) bound to the actin-cytoskeleton by cortical proteins, such as ZO-1,−2, and−3 (Capaldo et al., 2014).

The initial epithelial damage produced by *E. histolytica* is characterized by TJ opening, reflected as a dramatic drop of transepithelial electrical resistance (TEER) (Martínez-Palomo et al., 1985; Leroy et al., 2000; Betanzos et al., 2013), with the participation of PGE2 (Lejeune et al., 2011) and EhCPADH (Betanzos et al., 2013). PGE2 increases ion permeability by altering claudin-4 (Lejeune et al., 2011), while the EhCPADH complex affects claudin-1 and occludin (Betanzos et al., 2013). EhCPADH also damages adherens junctions (AJ) and desmosomes (DSM) (Hernández-Nava et al., 2017), structures that reinforce adhesion among epithelial cells, participate in cell polarity establishment and constitute centers of intracellular signaling (Capaldo et al., 2014).

The EhCPADH complex (Arroyo and Orozco, 1987), formed by an adhesin (EhADH) and a cysteine protease (EhCP112), participates in adhesion, cytolysis and phagocytosis of target cells (García-Rivera et al., 1999). EhCPADH, EhADH, and EhCP112 are secreted during trophozoite attack (Ocádiz et al., 2005; Bolaños et al., 2016). Moreover, an EhCP112 recombinant protein drops TEER of epithelial cells, and dislocates and degrades junctional molecules, including claudin-1, claudin-2, β-catenin, E-cadherin, desmoplakin-I/II and desmoglein-2 (Cuellar et al., 2017; Hernández-Nava et al., 2017).

EhADH contains a Bro1 domain (residues 9–349), characteristic of ALIX family members which are scaffold and multifunctional proteins (Odorizzi, 2006; Morita et al., 2007; Bissig and Gruenberg, 2014). Besides to its adhesive properties, EhADH is also an accessory protein of the endosomal sorting complex required for transport (ESCRT) machinery, whose components are pivotal players during phagocytosis in trophozoites (Avalos-Padilla et al., 2015, 2018). EhADH is localized at plasma membrane and endosomal compartments, and together with ESCRT members, contributes to multivesicular bodies formation (Bañuelos et al., 2012; Avalos-Padilla et al., 2015). Moreover, EhADH associates to cholesterol-trafficking proteins EhNPC1 and EhNPC2, suggesting an extra role in the uptake and transport of this essential lipid toward cellular membranes (Bolaños et al., 2016). Monoclonal antibodies (mAbAdh) against the C-terminal adherence domain (residues 480–600) of this protein (Montaño et al., 2017), inhibit trophozoite adhesion to and phagocytosis of erythrocytes, as well as destruction of MDCK cell monolayers (García-Rivera et al., 1999). However, the specific role of EhADH on epithelium damage has not been fully studied. What does the parasite protein do when it reaches the epithelium? Does it penetrate the target cell? If so, what does the adhesin carry out inside the cell? To approach these questions, we followed two different strategies: (i) we produced a recombinant protein (rEhADH) to scrutinize the effects of EhADH alone on epithelial cell monolayers, and (ii) we generated epithelial cells stably transfected with the *ehadh* gene (MDCK-EhADH) to evaluate EhADH effects within the cells.

Our findings showed rEhADH reaching the intercellular space of epithelial cells, co-localizing at TJ with claudin-1 and occludin. This protein was mainly internalized by clathrin-coated vesicles to MDCK cells. Meanwhile, MDCK cells expressing EhADH, exhibited epithelial aggregation and an increased adhesion to erythrocytes. Furthermore, EhADH mainly altered the amount of claudin-1 and occludin, reflected as an increase of TEER. Interestingly, we found that MDCK-EhADH cells resulted more susceptible to live trophozoites during TEER and cytopathic assays, than control cells. Thus, we suspect that EhADH, which preserved its adhesive properties within MDCK-EhADH cells, could be in some way preparing to epithelial cells for the trophozoites attack mediated by other virulence factors, whose identity and role on the concerted mechanism of epithelium damage should be further addressed.

## Materials and methods

### Antibodies

For EhADH immunodetection, we obtained rabbit polyclonal antibodies (α-EhADH) against a specific EhADH peptide (N-566 QCVINLLKEFDNTKNI 582-C) localized within the adherence domain. New Zealand male rabbits were immunized three times (each 2 weeks) with 300 μg of this peptide diluted in TiterMax® Gold Adjuvant liquid (Sigma). Other primary antibodies used were: mouse against EhADH (mAbAdh) (Arroyo and Orozco, 1987), α-actin (kindly donated by Dr. José Manuel Hernández from Department of Cellular Biology, CINVESTAV, Mexico), α-claudin-1 (Invitrogen), α-occludin (Invitrogen) and α-caveolin-1 (Santa Cruz Biotechnology); rabbit α-ZO-2 (Invitrogen), α-ZO-1 (Invitrogen), α-occludin (Invitrogen), α-α/β tubulin (Cell Signaling), and α-PCNA (Azuara-Liceaga et al., 2018); and goat α-clathrin (Santa Cruz Biotechnology) and α-GAPDH (Santa Cruz Biotechnology). For some experiments, mouse IgM isotype control (Thermo Fisher) was used. Secondary antibodies included: α-rabbit, α-mouse and α-goat HRP-labeled IgG (1:10,000) (Life technologies); and α-rabbit, α-mouse and α-goat FITC-, TRITC- and Cy5-labeled IgM, and IgG (1:100) (Zymed) antibodies.

### Cell cultures

Trophozoites of *E. histolytica* strain HM1:IMSS clone A (Orozco et al., 1983) were axenically cultured at 37°C in TYI-S-33 medium and harvested during the logarithmic growth phase by chilling the culture tubes for 10 min in an ice-water bath. Then, trophozoites were collected by centrifugation at 360 × g for 5 min (Diamond et al., 1978).

Madin Darby canine kidney (MDCK) epithelial cells type I (Cereijido et al., 1978) and human colorectal adenocarcinoma (Caco-2) from the C2BBe1 lineage (Sambuy et al., 2005) were grown in DMEM medium (Gibco) supplemented with 100 IU/ml penicillin (*in vitro*), 100 mg/ml streptomycin (*in vitro*), 10% fetal bovine serum (Gibco), and 0.08 U/ml rapid-acting insulin (Eli Lilly), at 37°C in a 95% air and 5% CO_2_ atmosphere (Betanzos et al., 2013). Transfected MDCK cells were cultured in DMEM medium supplemented with 0.5 mg/ml G-418 (Gibco), a neomycin derivative.

### Construction of plasmids and production of rEhADH

A DNA fragment of 2061 bp encoding the full-length of *E. histolytica ehadh* gene, was PCR amplified from the *pExEhNeo-Ehadh112* plasmid (Bañuelos et al., 2005) using the oligonucleotides described in Table 1. The *ehadh* gene was cloned into the *pGEX6P1* and *pcDNA3* plasmids (Invitrogen) between *BamHI* and *Xho1*, or *KpnI* and *BamH1* digestion sites, respectively. *Escherichia coli* C43 (DE3) and -DH5α bacteria (Invitrogen) were transformed with *pGEX6P1-ehadh* and *pcDNA3-ehadh*, respectively. Plasmids were purified by an affinity column (Qiagen) and automatically sequenced to corroborate the *ehadh* gene sequence.

Table 1Oligonucleotide sequences.

**Table:** 

**Complete gene**		**Sequence primer**	**TM (°C)**	**Restriction enzyme**	**Plasmid**
*ehadh*	F	GGGGTACCTATGAATAGACAATTCATTCCTGAA	69	*KpnI*	*pGEX6P1*
	R	CGGGATCCTTAAAGAGATGGAAACATAGGATTG		*BamH1*	
*ehadh*	F	CGGGATCCATGAATAGACAATTCATTCCT	72	*BamH1*	*pcDNA3*
	R	AGCTCGAGTTAAAGAGATGGA		*Xho1*

**Table:** 

**RT-PCR**		**Sequence primer**	**TM (**°**C)**	**Amplicon size (bp)**
*ehadh*	F	CATACCAATGAGAAAGTCAGATCC	65	796
	R	C GAGCACATCCTAACGCTAAGG		
*neo*	F	ATGATTGAACAAGATGG	50	650
	R	TTAGAAGAACTCGTC		
*gapdh*	F	TCCTGCACCACCAACTGCTT	60	100
	R	GGCATGGACGGTGGTCATGA		

*Restriction enzyme sites are underlined. F, forward; R, reverse*.

To produce an EhADH recombinant protein (rEhADH), *E. coli* C43 (DE3) bacteria were transformed with the *pGEX6P1-ehadh* plasmid. The recombinant protein was induced with 1 mM IPTG and purified as described (Bañuelos et al., 2012).

### Transfection assays

Transfection of *pcDNA3-ehadh* or *pcDNA3* plasmids into MDCK cells was performed with the Lipofectamine® 2,000 transfection reagent (Invitrogen), following the manufacturer's instructions. Positive clones were selected after 48 h transfection using 1 mg/ml of G-418 in the culture medium. After 3 weeks, the antibiotic was diminished to 0.5 mg/ml.

### RT-PCR experiments

Total RNA from non-transfected and transfected MDCK cells was isolated by TRIzol® Reagent (Invitrogen). cDNAs were reverse-transcribed from 1 μg of total RNAs using M-MLV Reverse Transcriptase (Promega) and following the manufacturer's instructions. Then, PCR for *ehadh, neo*, and *gapdh* (as internal control) genes were performed with the oligonucleotides described in Table 1. PCR amplifications were done following standard procedures for cycling conditions that included an initial denaturing step at 94°C for 1 min, followed by 30 cycles of 94°C for 1 min, 50, 60, or 65°C (according to respective Tm) for 1 min, and 72°C for 1 min, with a final extension step at 72°C for 7 min. Products were separated by electrophoresis in 1% agarose gels and revealed by ethidium bromide staining (Bolaños et al., 2016).

### Cellular extracts

Bacteria were lysed with 2% sarcosyl and 0.5% Triton X-100 in PBS by sonication at 4°C.

Trophozoites were washed twice with ice-cold PBS (140 mM NaCl, 2.7 mM KCl, 10 mM Na_2_HPO_4_, 1.8 mM KH_2_PO_4_, pH 7.4) and lysed by freeze-thawing in the presence of 100 mM p-hydroxymercuribenzoate (PHMB) and 40 μg/ml of E-64 (García-Rivera et al., 1999).

MDCK cells were collected with a rubber policeman, washed three times with ice-cold PBS and lysed for 30 min in RIPA buffer (40 mM Tris-HCl pH 7.6, 150 mM NaCl, 2 mM EDTA, 10% glycerol, 1% Triton X-100, 0.5% sodium deoxycholate, 0.2% SDS, 1 mM PMSF and the Complete^TM^ [Roche] protease inhibitor cocktail) under continuous and vigorous shaking. Extracts were sonicated three times for 30 s and centrifuged for 15 min at 25,000 × g to eliminate undissolved cellular debris (Betanzos et al., 2013).

### Western blot assays

Protein samples were separated by 6, 8, 10, or 15% sodium dodecyl sulfate polyacrylamide gel electrophoresis (SDS-PAGE), subsequently transferred onto nitrocellulose membranes and incubated 1 h with 5% non-fat milk. Blotting were performed overnight (ON) with mouse mAbAdh (1:50), α-claudin-1 (1:1,000) or α-actin (1:1,500); rabbit α-EhADH (1:3,000), α-occludin (1:1,000), α-ZO-1 (1:500), α-ZO-2 (1:800) or α-tubulin (1:3,000); or goat α-GAPDH (1:10,000) antibodies. Then, we used HRP-conjugated secondary antibodies against mouse IgM and IgG, rabbit IgG or goat IgG, followed by a chemiluminiscence detection system (ECL-Plus kit; Amersham Pharmacia Biotech). Protein bands were visualized on a MicroChemi System (DNR Bio-Imaging), and densitometry analysis were performed using the ImageJ software.

### Interaction of epithelial cells with recombinant proteins

Confluent and sparse MDCK and Caco-2 cell monolayers were apically incubated with 10 μg/cm^2^ of rEhADH or rEhPCNA (Cardona-Felix et al., 2011) for different times at 37°C. After interaction, epithelial cells were washed five times with PBS (140 mM NaCl, 2.7 mM KCl, 10 mM Na_2_HPO_4_, 1.8 mM KH_2_PO_4_, pH 6.8) to eliminate unbound molecules, and treated for immunofluorescence assays as described below.

In the case of rEhADH, it was Alexa Fluor 647-labeled using the Antibodies Labeling Kit (Molecular Probes), following the manufacturer's instructions. Briefly, 100 μg of rEhADH were equilibrated with 0.1 M sodium bicarbonate to allow the adequate succinimidyl ester reaction with primary amines of the protein, to form stable dye-protein conjugates (Cuellar et al., 2017).

### Immunofluorescence assays

MDCK and Caco-2 cell monolayers grown on glass coverslips were fixed and permeabilized with absolute ethanol for 30 min at −20°C. Cells were blocked for 30 min with 0.5% BSA and 0.05% saponin, and then incubated ON at 4°C with mouse α-claudin-1 (1:25), mouse α-occludin (1:1,000), rabbit α-EhADH (1:100), rabbit α-ZO-1 (1:100) or rabbit α-ZO-2 (1:100) antibodies. For co-localization experiments, monolayers were incubated with mouse mAbAdh (1:10), rabbit α-EhADH (1:100) or rabbit α-PCNA (1:100), and mouse α-caveolin-1 (1:100), goat α-clathrin (1:100), mouse α-claudin-1 (1:25), mouse α-occludin (1:1,000), rabbit α-ZO-1 (1:100) or rabbit α-ZO-2 (1:100) antibodies. After three-times washing with PBS, preparations were accordingly incubated for 1 h at room temperature (RT) with FITC-, TRITC- and Cy5-conjugated secondary antibodies. For cells in suspension, the procedure was performed in Eppendorf tubes and finally placed on coverslips. In some cases, nuclei were counterstained with 2.5 μg/ml of 4′,6-diamidino-2-phenylindole (DAPI) (Zymed) during 5 min. Preparations were mounted with the antifade reagent Vectashield (Vector laboratories) and examined through a confocal microscope (Leica TCS_SP5_MO) in *Z*-stack optical sections of 0.5 μm and *xz*- and *zy*-planes. In all cases, 10 fields per condition were analyzed and representative images were selected for each time.

For inhibition of clathrin-coated vesicles transport, MDCK cells were pre-incubated with 300 mM sucrose diluted in DMEM medium at 37°C for 1 h or DMEM medium as a control (Mosso et al., 2008). Then, cells were processed for immunofluorescence as above.

### Aggregation and adhesion assays

Transfected MDCK cells were trypsinysed, washed twice in PBS, diluted in DMEM and suspended as hanging drops from the lid of a 24 well culture dish. Wells were filled with sterile water to prevent drops drying (Thoreson et al., 2000). Culture dishes were kept in a humid 5% CO_2_ incubator at 37°C and cell aggregation was determined 4 h after plating. Cells in each drop were passed 10 times through a standard 200 μl Gilson pipet tip and photographed through a Nikon E600 microscope, using a 20X phase contrast objective. The number of isolated cells and cells forming aggregates were counted in five random fields from three independent experiments.

Adhesion assays were lightly modified from previously described (Orozco et al., 1983). Briefly, MDCK cells in suspension were mixed with washed erythrocytes (1:100 ratio). Cell mixture was incubated for 0.5, 1 and 2 h at 37°C, fixed with 2.5% glutaraldehyde for 30 min at 37°C and washed three-times with PBS. Erythrocytes were counterstained with 4.5 mM diaminobenzidine for 30 min at 37°C. Finally, adhered erythrocytes to each MDCK cell in ten random fields from three independent experiments were counted through a Nikon E600 microscope.

For inhibiting cell aggregation and erythrocytes adhesion, before experiments, 2 × 10^5^ MDCK cells were incubated with 10 μg mAbAdh antibody or IgM isotype for 30 min at 37°C.

### Measurement of transepithelial electrical resistance (TEER)

Transfected MDCK cells were seeded on Transwell filter supports (6.5 mm diameter and 0.4 μm pore; Corning). Three days after plating, and after confirming through an inverted microscope that monolayers reached confluency, the TEER was measured using an EVOM epithelial voltmeter (World Precision Instruments) (Betanzos et al., 2013). TEER values were obtained by subtracting cell-free filter readings.

For some experiments, transfected MDCK cells were apically incubated with live trophozoites (10^5^/cm^2^) and TEER was monitored during 90 min.

### Paracellular flux assays

TRITC-dextran (3 mg/ml) of 4 kDa (Sigma Aldrich) was added to the apical side of transfected epithelial cells in confluency, seeded in Transwell filters. After 90 min incubation at 37°C with gentle shaking and darkness, samples from the basal chamber were collected and the diffused fluorescent tracer was measured in a fluorimeter (excitation λ = 547 nm; emission λ = 572 nm). Emission values were converted to TRITC-dextran concentration, using a standard curve (Cuellar et al., 2017). As a positive control, before tracer addition, cells were incubated for 30 min with 5 mM EDTA.

### Cytopathic assays

Transfected MDCK cells in confluency, seeded in 24-well plates, were twice-washed with PBS to remove traces of serum and then incubated with live trophozoites (50, 100, and 250 × 10^3^) suspended in TYI-S-33 medium without serum. Incubation was carried out for 2 h at 37°C in a CO_2_ containing incubator. The reaction was stopped by cooling cell culture plates in an ice-water bath, to release adhered trophozoites. Epithelial cells were carefully washed with cold PBS and monolayer destruction was measured as described (Bracha and Mirelman, 1984).

### Statistical analysis

All data shown in this work, were representative from three independent experiments performed at least by triplicate. Results were displayed as mean and standard error. For statistical analysis, we followed the two-tailed Student *t*-test and two-ways ANOVA using the GraphPad Prism 5 software. Statistical significance was assumed when ^*^p < 0.05, ^**^p < 0.01, or ^***^p < 0.001.

### Ethics statement

The Centre for Research and Advanced Studies (CINVESTAV) fulfilled the standard of the Mexican Official Norm (NOM-062-ZOO-1999) and Technical Specifications for the Care and Use of Laboratory Animals based on the Guide for the Care and Use of Laboratory Animals The Guide, 2011, NRC, USA with the Federal Register Number BOO.02.03.02.01.908. This is awarded by the National Health Service, Food Safety and Quality (SENASICA) belonging to the Animal Health Office of the Secretary of Agriculture, Livestock, Rural Development, Fisheries and Food (SAGARPA), an organization that verifies the state compliance of such Mexican Official Norm (NOM) in Mexico. The Institutional Animal Care and Use Committee (IACUC/ethics committee) of CINVESTAV, as the regulatory office for the approval of research protocols involving the use of laboratory animals and, in fulfillment of the NOM, has reviewed and approved all animal experiments (Protocol Number 0505-12, CICUAL 001).

## Results

### Recombinant EhADH (rEhADH) penetrates epithelial cells through the paracellular route

**Figure 1:**
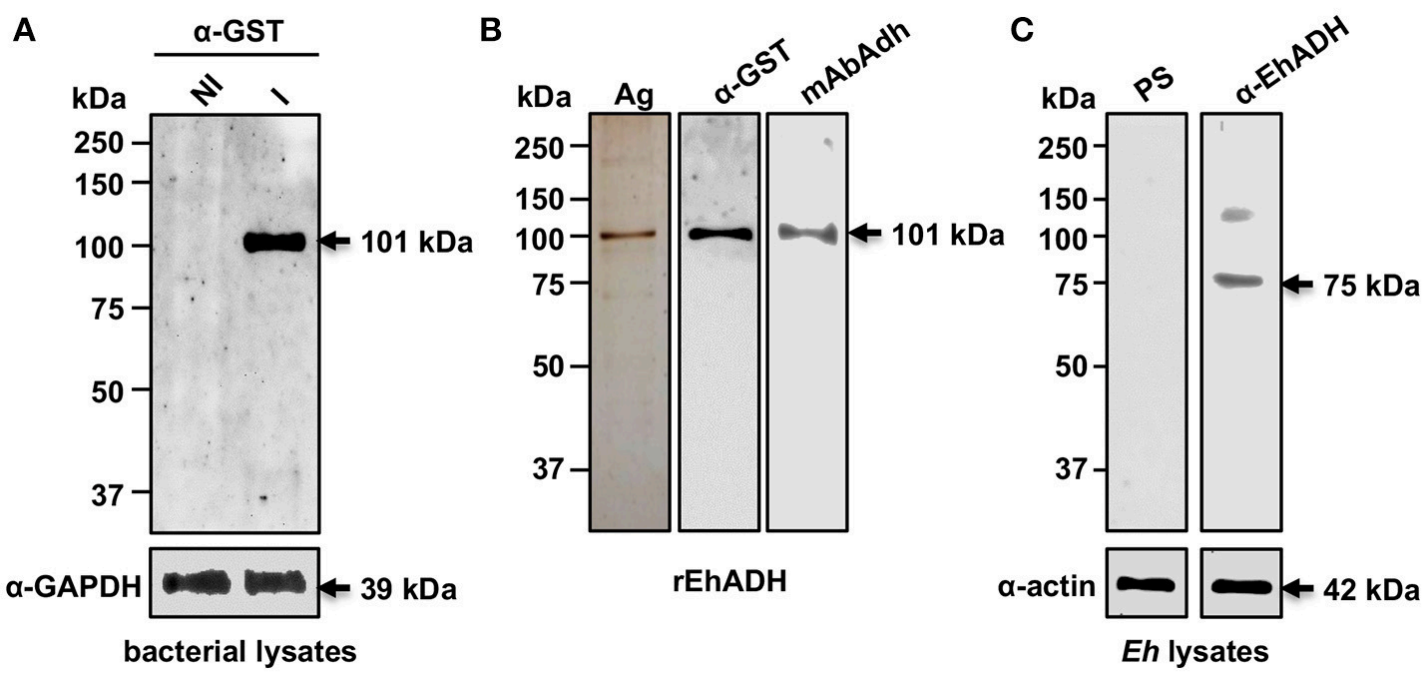
Production of rEhADH. **(A)** Lysates of *E. coli* C43 (DE3) bacteria transformed with the *pGEX6P1-ehadh* plasmid. The recombinant GST-EhADH (rEhADH) expression was induced in bacteria by 1 mM IPTG and analyzed by western blot assays using the α-GST or α-GAPDH (loading control) antibodies. NI: non-induced. I: induced. **(B)** rEhADH purity was analyzed by silver gel staining (Ag) and western blot assays using α-GST and mAbAdh antibodies. **(C)** Trophozoites (*Eh*) lysates were analyzed by western blot using pre-immune serum (PS), α-EhADH or α-actin (loading control) antibodies. Numbers at left: molecular weight standards. Arrows: expected molecular weight of proteins.

**Figure 2:**
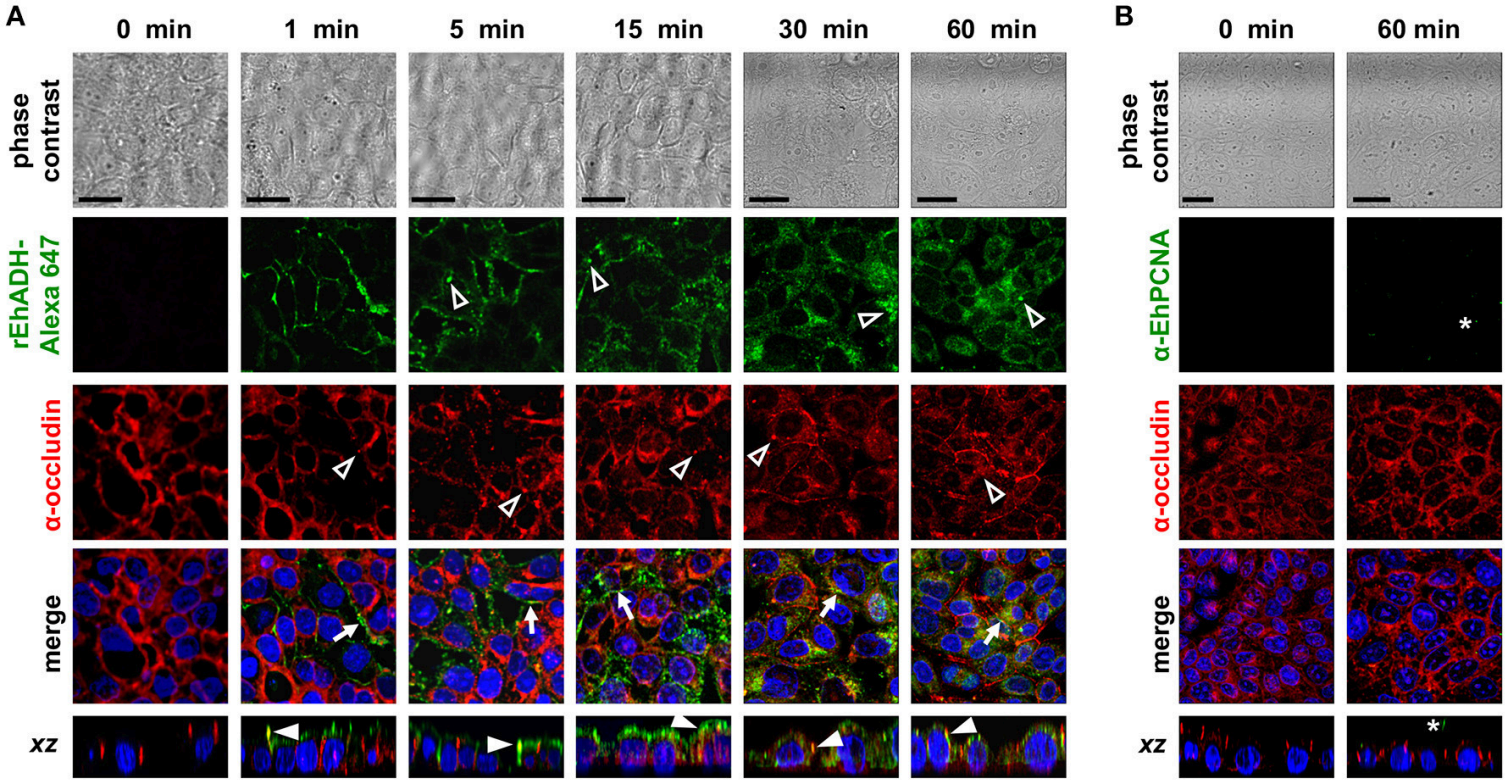
Co-localization of rEhADH with occludin on MDCK cells. **(A)** rEhADH coupled to Alexa 647 (green) or **(B)** rEhPCNA were apically added to confluent MDCK cells and incubated for different times, then, cells were fixed and processed for immunofluorescence assays using the α-occludin antibody (red). rEhPCNA was detected by α-EhPCNA antibody (green). Nuclei were counterstained with DAPI (blue) and preparations were analyzed through a confocal microscope at *xy-* and *zy-*planes. Arrows: co-localization at cellular borders. Full arrowheads: co-localization at lateral membrane. Empty arrowheads: localization of EhADH (green) and occludin (red) at cytoplasm. Asterisks: EhPCNA localization. Bar = 20 μm.

**Figure 3:**
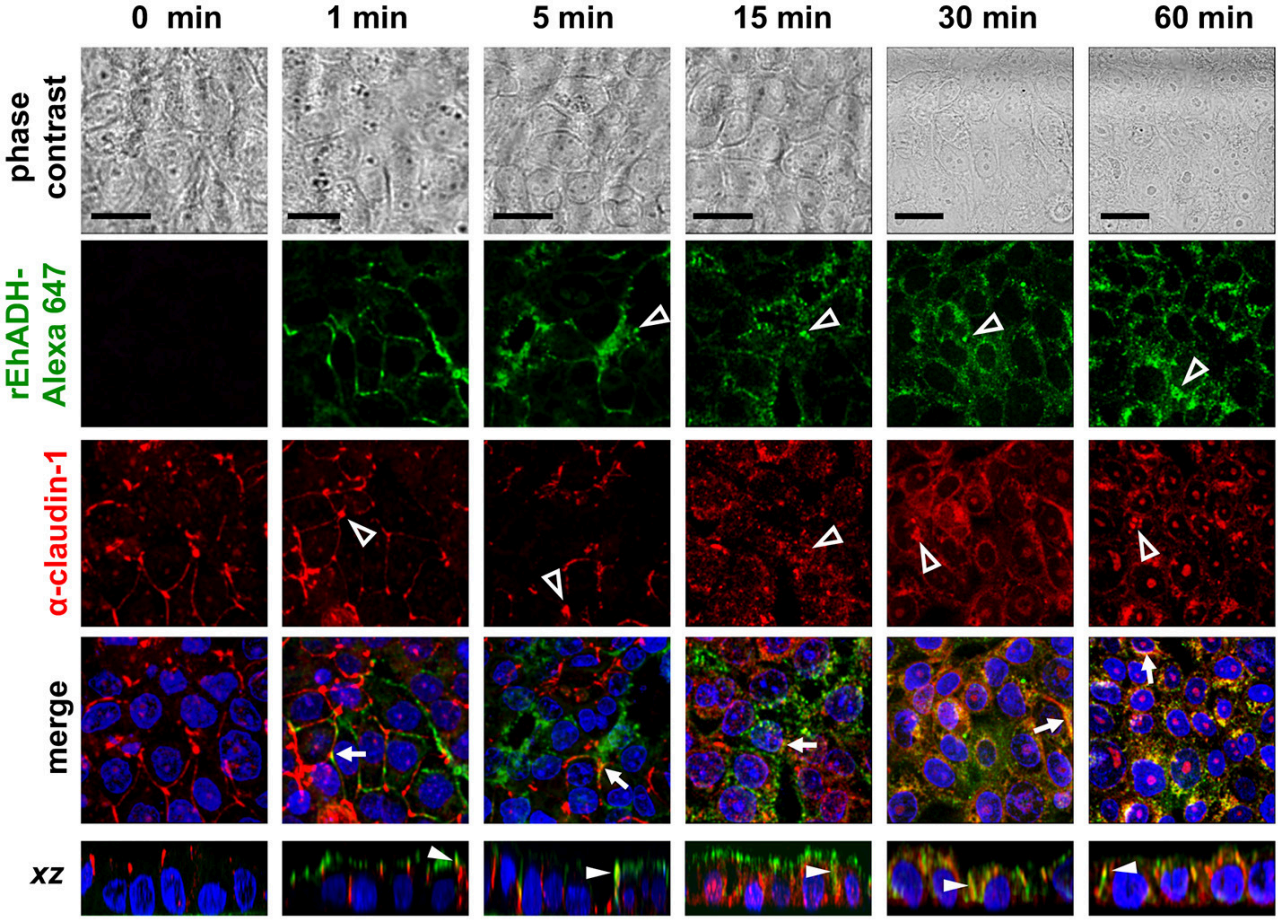
Co-localization of rEhADH with claudin-1 on MDCK cells. rEhADH coupled to Alexa 647 (green) was apically added to confluent MDCK cells and incubated for different times, then, cells were fixed and processed for immunofluorescence assays using the α-claudin-1 antibody (red). Nuclei were counterstained with DAPI (blue) and preparations were analyzed through a confocal microscope at *xy-* and *zy-*planes. Arrows: co-localization at cellular borders. Full arrowheads: co-localization at lateral membrane. Empty arrowheads: localization of EhADH (green) and claudin-1 (red) at cytoplasm. Bar = 20 μm.

**Figure 4:**
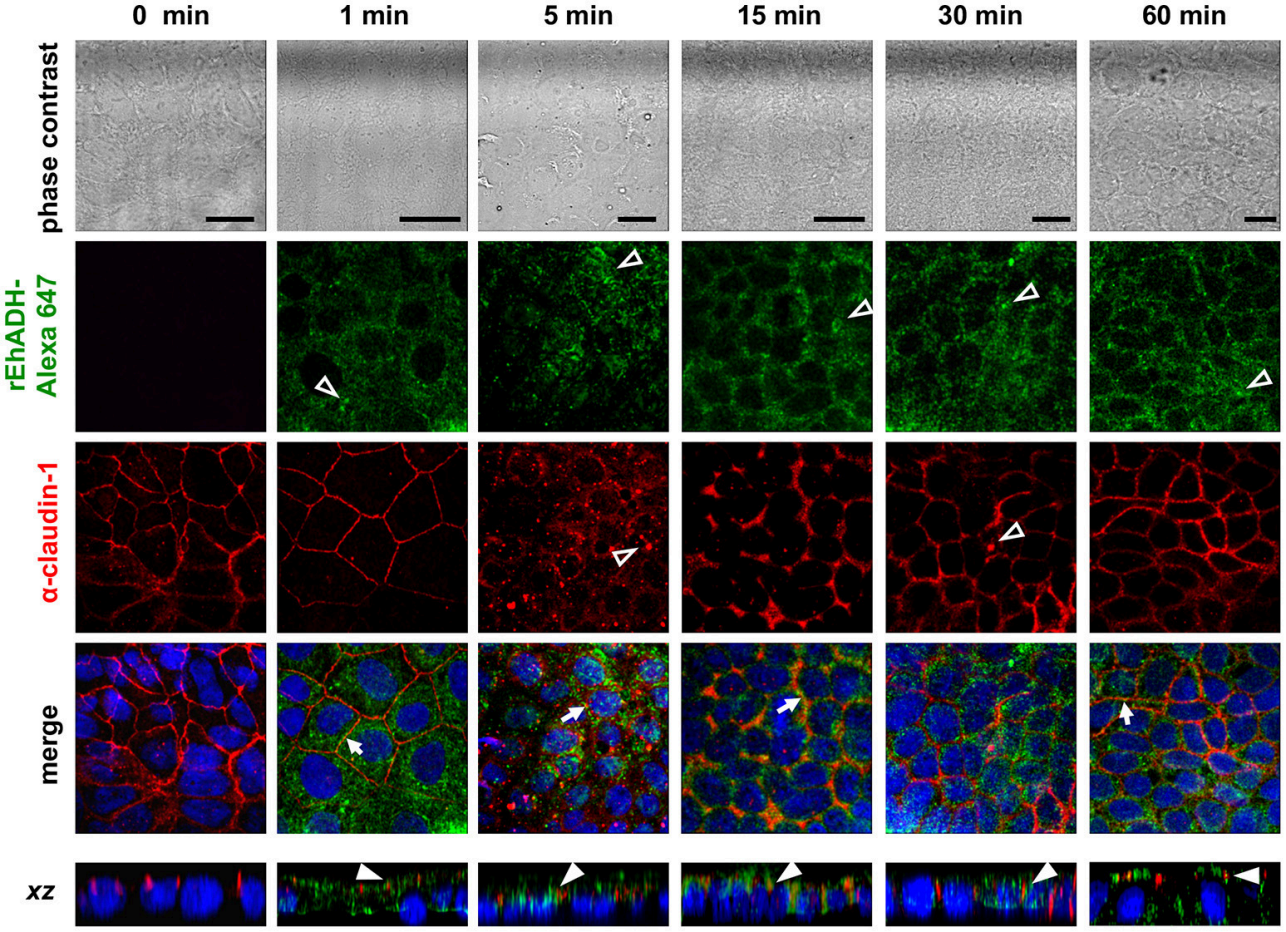
Co-localization of rEhADH with claudin-1 on Caco-2 cells. rEhADH coupled to Alexa 647 (green) was apically added to confluent Caco-2 cells and incubated for different times, then, cells were fixed and processed for immunofluorescence assays using the α-claudin-1 antibody (red). Nuclei were counterstained with DAPI (blue) and preparations were analyzed through a confocal microscope at *xy-* and *xz-*planes. Arrows: co-localization at cellular borders. Full arrowheads: co-localization at lateral membrane. Empty arrowheads: localization of EhADH (green) and claudin-1 (red) at cytoplasm. Bar = 20 μm.

The EhCPADH complex is a virulence factor involved in adhesion, phagocytosis and cytolysis of target cells by *E. histolytica* trophozoites. This protein, as well as EhCP112 and EhADH, are secreted during host invasion, and from the medium they reach the target cell surface (García-Rivera et al., 1999; Ocádiz et al., 2005). To investigate the role of EhADH on epithelium damage without the interference of other trophozoite proteins, we produced an EhADH recombinant protein (rEhADH). The *ehadh* gene was cloned into the *pGEX6P1* plasmid, and *pGEX6P1-ehadh* transformed bacteria were induced for EhADH expression as a recombinant protein tagged to GST (Figure 1A). After purification by a glutathione-sepharose resin, the purity of rEhADH was visualized in silver-stained gels. A single 101 kDa band corresponding to the expected molecular weight for EhADH (75 kDa) (García-Rivera et al., 1999) plus GST (26 kDa) was evidenced (Figure 1B). The identity of the recombinant protein was probed by western blot assays using α-GST and mAbAdh antibodies. Both antibodies recognized the same 101 kDa band (Figure 1B); whereas in trophozoites lysates, the polyclonal α-EhADH antibody directed against a EhADH polypeptide (residues 566–582) located within the adherence domain, recognized the 75 kDa band (Figure 1C). Purified rEhADH coupled to the Alexa 647 fluorescent dye, was added to the apical side of confluent MDCK cells. Confocal microscopy images showed that immediately after rEhADH addition, the protein located at cellular borders, then, it appeared co-localizing with occludin and claudin-1 at the TJ region (Figures 2, 3). *xz*-planes images revealed that rEhADH was posed firstly on the apical surface of cells. Next, it penetrated through the intercellular space to be later found inside the cells. During these experiments, monolayers seemed intact under phase contrast images, even at 60 min rEhADH incubation. However, confocal images revealed that after cells contact with rEhADH (1 and 5 min), occludin and claudin-1 were delocalized from the cellular borders and eventually, both proteins were internalized to the cytoplasm (15 min). Afterwards, at 30 and 60 min these proteins partially recovered their TJ localization. Another *E. histolytica* recombinant protein, rEhPCNA (Cardona-Felix et al., 2011), did not bind to epithelial cells and neither produced modifications on TJ proteins (Figure 2B), indicating that the rEhADH effects were specific. We also investigated the effect of rEhADH on sparse MDCK cultures, where TJ structure and functions are not yet well stablished. Results evidenced claudin-1 at cellular borders in a discontinuous pattern, and this protein was not diminished neither internalized during all rEhADH incubation times (Figure S1). Whilst, at the beginning of incubation, rEhADH was mainly localized at the borders of cellular groups in growth. Nevertheless, at 30 min incubation, rEhADH was found at plasma membrane and cytoplasm (Figure S1), in accordance to the effect of this protein on confluent cells at 15 min (Figure 3). To analyse the effect produced by rEhADH on the natural colonization site in the host, we assessed its localization on human intestinal Caco-2 cells. Confocal images showed similar results in Caco-2 (Figure 4) and MDCK cells, confirming that rEhADH contacts epithelial cells, then displaces along the intercellular space, penetrates the cells and delocalizes occludin and claudin-1.

### rEhADH is internalized by clathrin-coated vesicles

**Figure 5:**
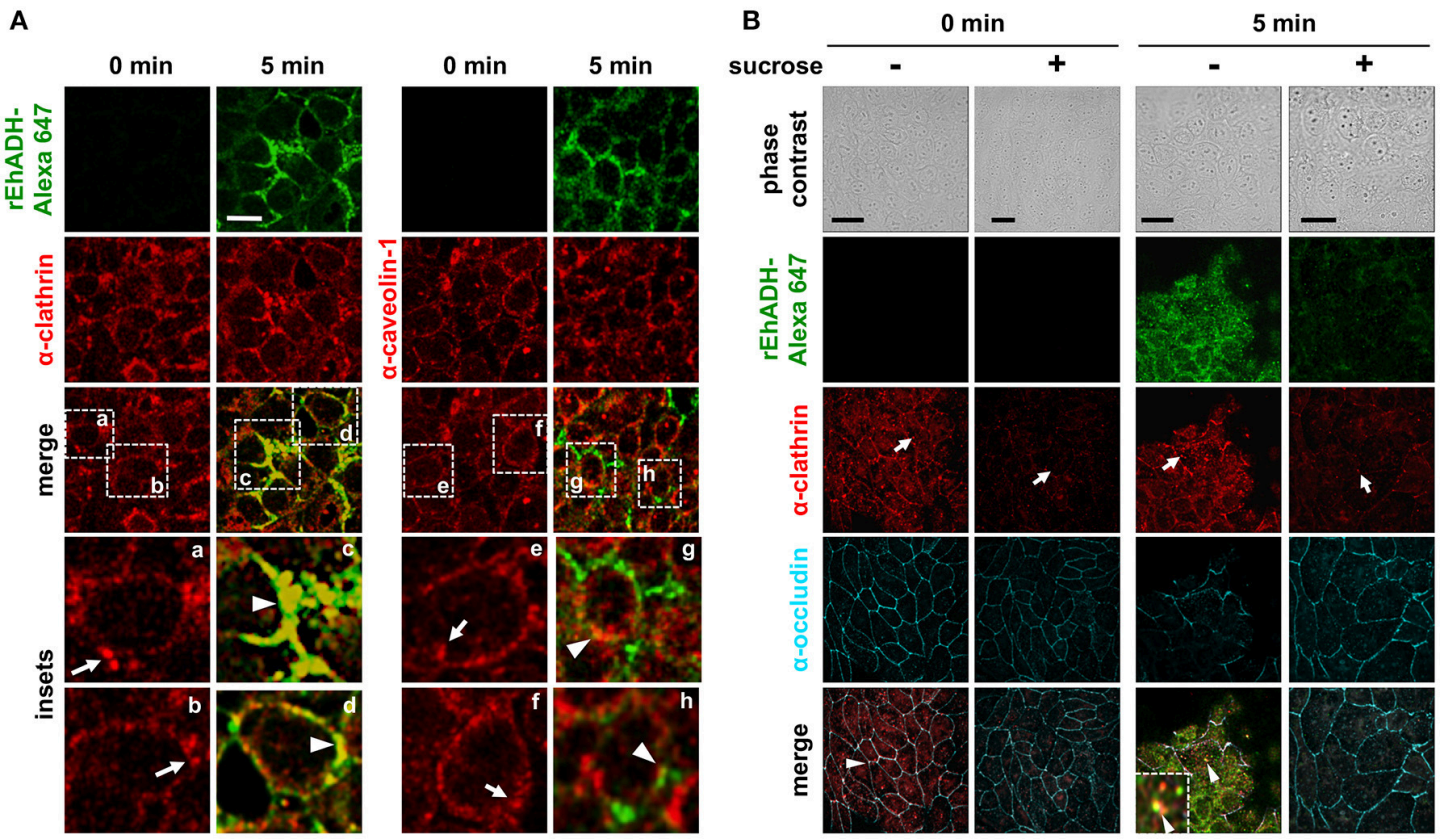
Co-localization of rEhADH with clathrin and caveolin-1 on MDCK cells. **(A)** rEhADH coupled to Alexa 647 (green) was apically added to confluent MDCK cells and incubated for 5 min, then, cells were fixed and processed for immunofluorescence assays using α-clathrin or α-caveolin-1 antibodies (red). Preparations were analyzed through a confocal microscope at the *xy-*plane. Insets: magnification of squares (a–h) from merge images. **(B)** MDCK cells were incubated for 1 h with DMEM medium (–) or 300 mM sucrose (+) at 37°C and then processed as in **(A)**, but also using the α-occludin antibody (cyan). Inset: magnification of rEhADH and clathrin co-localization. Arrows: vesicles close to cellular borders. Arrowheads: co-localization. Bar = 20 μm.

Some pathogens and their secreted molecules enter the host cell by lipid microdomains known as caveolae and clathrin-coated vesicles (Rosenberger et al., 2000; Moreno-Ruiz et al., 2009; Machado et al., 2012). In fact, we have reported that EhCP112, the other part of the EhCPADH complex, is introduced to epithelial cells by these kind of vesicles (Hernández-Nava et al., 2017). To explore whether rEhADH was internalized into MDCK cells through any of these mechanisms, we used antibodies against human clathrin−1 and caveolin. MDCK cells were incubated with fluorescence-labeled rEhADH and followed by confocal microscopy. Images evidenced that rEhADH strongly localized at clathrin-coated vesicles, whereas its localization at caveolae was poor (Figure 5A). Next, to confirm rEhADH endocytosis mediated by clathrin-coated vesicles, we used sucrose to inhibit this type of transport (Mosso et al., 2008). MDCK cells treated for 1 h with sucrose diminished clathrin-coated vesicles (Figure 5B). These cells now incubated for 1 (Figure S2) and 5 min (Figure 5B) with rEhADH, poorly bound it and were not able to internalize this protein, while sucrose-untreated cells presented rEhADH at cellular borders and inside the cells, co-localizing with clathrin (Figure 5B). In rEhADH-untreated MDCK cells, the sucrose treatment also affected the occludin pattern at TJ region, lightly reducing its localization at cellular borders. As mentioned above, in sucrose-untreated MDCK cells and incubated with rEhADH, occludin was delocalized (Figure 2A), however after 5 min rEhADH incubation, when clathrin-transport is inhibited, occludin remained in its characteristic localization (Figure 5B). These findings robustly suggested that rEhADH is internalized to target cells by clathrin-coated vesicles.

### *E. histolytica* EhADH is expressed by *pcDNA3-ehadh* transfected MDCK cells

**Figure 6:**
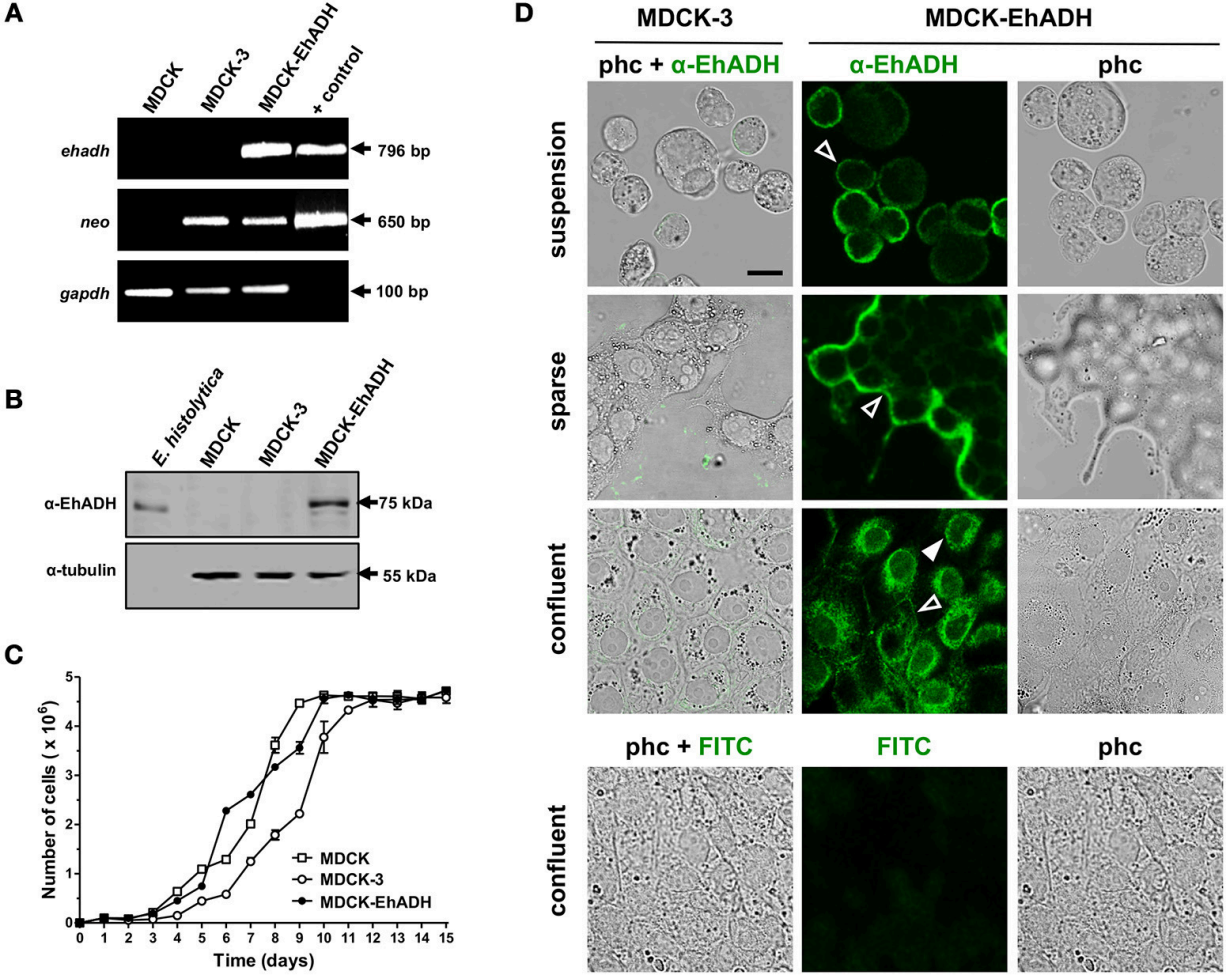
Expression of the *E. histolytica* EhADH protein in MDCK epithelial cells. MDCK cells were transfected with the *pcDNA3* (MDCK-3) or *pcDNA3-ehadh* (MDCK-EhADH) plasmids and EhADH expression and localization were analyzed. **(A)** RT-PCR assays of non-transfected (MDCK) or transfected MDCK cells, using specific primers for *ehadh, neo* and *gapdh* (as internal control) genes. As positive control, the *pcDNA3-ehadh* plasmid was used as template in PCR experiments. Numbers at right: amplicon size. **(B)** Western blot experiments of epithelial lysates, employing α-EhADH and α-tubulin (as loading control) antibodies. Trophozoites lysates were used as positive control. Numbers at right: expected molecular weight of proteins. **(C)** Growth curves of MDCK cells. After 48 h of plasmid transfection into cells, growth medium was supplemented with 1 mg/ml G-418, whereas non-transfected cells were grown in normal medium. The number of cells were counted in a Neubauer haemocytometer. Each point represents the mean and standard error of three independent experiments. **(D)** Suspension, sparse and confluent transfected MDCK cells were fixed, permeabilized and processed for immunofluorescence assays using the mAbAdh antibody. Bottom panel: MDCK-EhADH cells treated only with the secondary antibody (FITC). All images represent *xy*-optical sections. Empty arrowheads: plasma membrane. Full arrowhead: perinuclear localization. phc: phase contrast. Bar = 20 μm.

To study the effect produced by EhADH inside epithelial cells, we expressed this protein in MDCK cells that were transfected with the *pcDNA3-ehadh* plasmid, which contains a promoter for expressing exogenous genes in mammalian cells. Stably transfected cells were selected with G-418. To evidence the presence of the *ehadh* transcript, we performed RT-PCR assays. Results revealed that only MDCK cells transfected with *pcDNA3-ehadh* (MDCK-EhADH), expressed the *ehadh* transcript, in contrast to *pcDNA3* transfected (MDCK-3) or non-transfected (MDCK) cells (Figure 6A). The stable gene transfection in these cells was also demonstrated by RNA expression of the *neomycin* resistance gene, which was also expressed by MDCK-3 cells. The expression of the EhADH protein was verified by western blot assays, using total extracts of MDCK-EhADH cells and a specific polyclonal antibody against the adhesin (α-EhADH). The α-EhADH antibody recognized a single 75 kDa band (Figure 6B), as reported (García-Rivera et al., 1999), and did not detect any protein in MDCK-3 and non-transfected cells (Figure 6B). In general, growth rates for all cell types were similar (Figure 6C). Thus, MDCK cells constitutively expressed *E. histolytica* EhADH and apparently, by itself the adhesin did not affect the viability and growth of epithelial cells.

### EhADH is localized at the MDCK cellular membrane

Protein function is closely related to its location in cellular structures. In trophozoites, EhADH is localized at plasma membrane, endosomes and cytoplasm (Avalos-Padilla et al., 2015; Montaño et al., 2017). The location of the EhADH protein in MDCK-EhADH cells was studied at different stages of the cell monolayer formation through immunofluorescence assays using the mAbAdh antibody. In MDCK-EhADH cells in suspension, EhADH mainly appeared at the plasma membrane (Figure 6D). In sparse cultures, when cell polarization begins, EhADH was predominantly concentrated at borders of the growing cellular groups, where the cell monolayer was extending (Figure 6D). This pattern correlated with that observed in sparse non-transfected cultures incubated with rEhADH (Figure S1). Interestingly, MDCK-EhADH confluent monolayers presented EhADH at cellular borders (Figure 6D), which is in concordance with the localization of its rat homolog (Alix), present at cellular borders of immortalized epithelial cells from rat choroid plexus (Campos et al., 2016). EhADH was also abundantly detected around nuclei, probably in the endoplasmic reticulum, where adhesin is being synthesized (Figure 6D). As expected, EhADH was not present in MDCK-3 cells. These results indicated that as in trophozoites, EhADH localized at the plasma membrane and cytoplasm of transfected epithelial cells. Additionally, these experiments corroborated that EhADH stable expression did not modify the morphology of MDCK cells.

### EhADH induces aggregation of target cells

**Figure 7:**
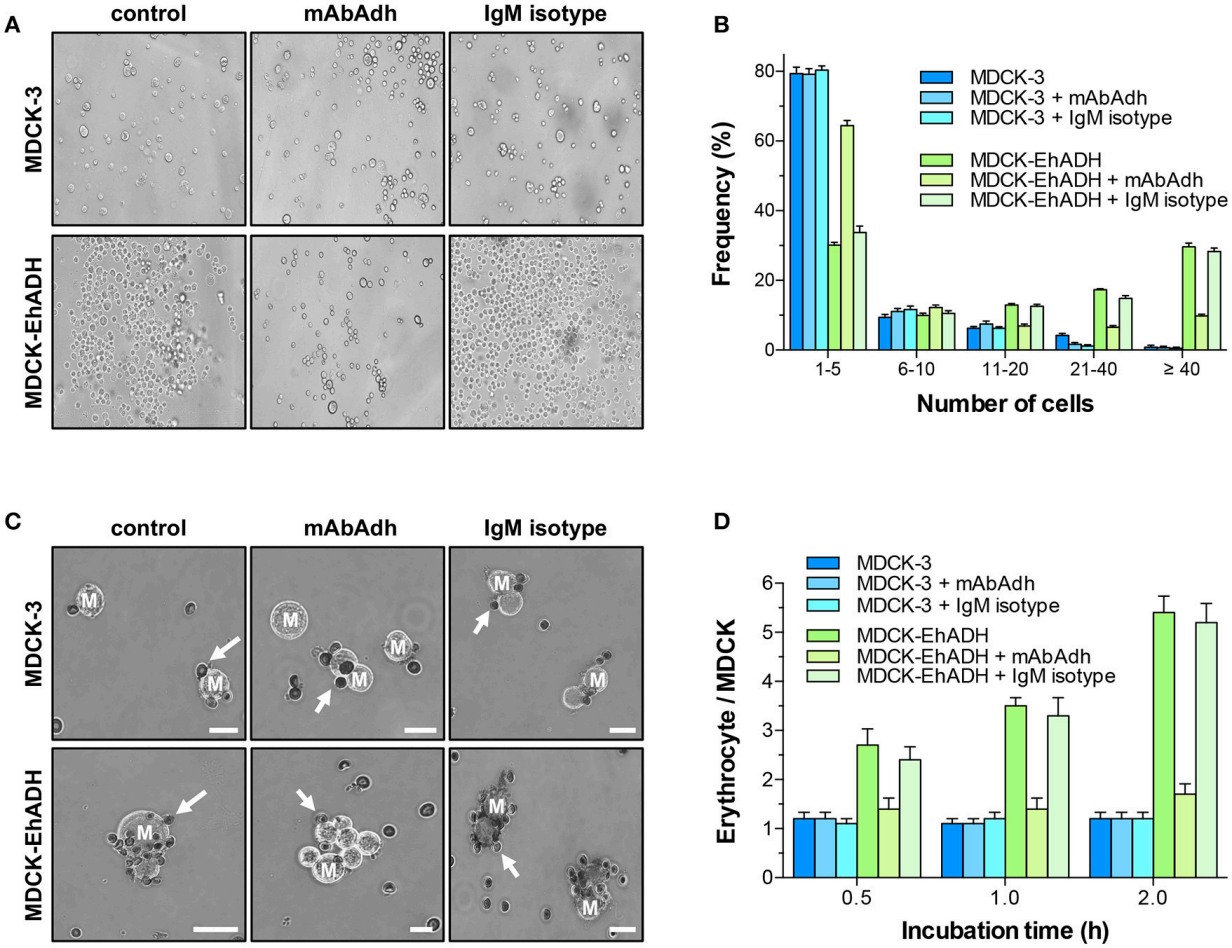
Adherence properties of MDCK-EhADH cells. **(A)** Transfected MDCK cells were trypsinysed, incubated with PBS (control), mAbAdh antibody or IgM isotype for 30 min at 37°C and suspended as hanging drops from the lid of a 24 well culture dish. Four hours after plating, cells in drops were resuspended and photographed in an optical microscope with 20X phase contrast objective (representative images). **(B)** Single cells and cellular clusters showed in **(A)** were counted, and frequency for each group with similar cell numbers were graphed. Data represent the mean and standard error of five fields quantified from three independent experiments. **(C)** Transfected MDCK cells were trypsinysed, incubated with PBS (control), mAbAdh antibody or IgM isotype for 30 min at 37°C, and mixed with erythrocytes. After 0.5, 1 and 2 h of incubation, erythrocytes were stained with diaminobenzidine and adhered erythrocytes (arrows) to MDCK cells (M) were photographed. Representative images from 2 h incubation are shown. Bar = 10 μm. **(D)** Adhered erythrocytes to each MDCK cell were counted. Values represent the mean and standard error from three independent experiments by triplicate. Differences found between groups were statistically significant (^***^p < 0.001), according to two-ways ANOVA test.

In *E. histolytica* trophozoites, EhADH is involved in target cell adherence (García-Rivera et al., 1999; Madriz et al., 2004; Martinez-Lopez et al., 2004). Therefore, we analyzed whether EhADH expressed by MDCK cells, evoked its adhesin function to target cells. For these experiments, transfected epithelial cells were trypsinysed and incubated in PBS at 37°C for 4 h, then, we counted the number of clumps formed and the number of cells contained in each one. Optical microscopy images clearly evidenced that MDCK-EhADH cells formed more clumps with a higher number of cells than MDCK-3 cells that did not express the adhesin (Figure 7A). For quantification, clumps were grouped according to the number of contained cells (1–5, 6–10, 11–20, 21–40, and more than 40 cells) and their frequencies were determined. Whilst MDCK-3 cells formed 82% of clumps containing 1–5 cells, only 43% of clumps were constituted by MDCK-EhADH cells (Figure 7B). In contrast, MDCK-EhADH and MDCK-3 cells formed 30% and 6% of clumps containing more than 40 cells, respectively. To confirm the adhesive role of EhADH in MDCK cells, before aggregation assays, transfected cells were pre-incubated with the mAbAdh antibody to inhibit clumps formation. Results showed that the antibody treatment, reduced the frequency of clumps with a higher number of cells in MDCK-EhADH, but not in MDCK-3 cells (Figure 7B). In contrast, an IgM isotype used as control, did not modify clumps frequency. These results suggested that MDCK-EhADH cells displayed stronger associations among them than MDCK-3 cells.

In trophozoites, EhADH also participates in erythrocytes adhesion (Avalos-Padilla et al., 2015; Bolaños et al., 2016). Thus, we studied whether MDCK-EhADH cells were able to bind erythrocytes. Transfected MDCK cells were incubated for different times with red blood cells and adhered erythrocytes were counted. Surprisingly, both types of transfected MDCK cells were able to attach erythrocytes (Figure 7C). However, after 1 and 2 h incubation, MDCK-EhADH cells adhered twice more erythrocytes than MDCK-3 cells (Figure 7D). To inhibit erythrocytes adhesion, again, before assays, transfected cells were pre-incubated with the mAbAdh antibody, resulting in a decreased number of red blood cells adhered to MDCK-EhADH, but not to MDCK-3 cells (Figure 7D). The IgM isotype presented a similar effect than in control transfected MDCK cells. These experiments showed that EhADH preserved its adhesive properties within MDCK-EhADH cells.

### MDCK-EhADH cells present an increased teer

**Figure 8:**
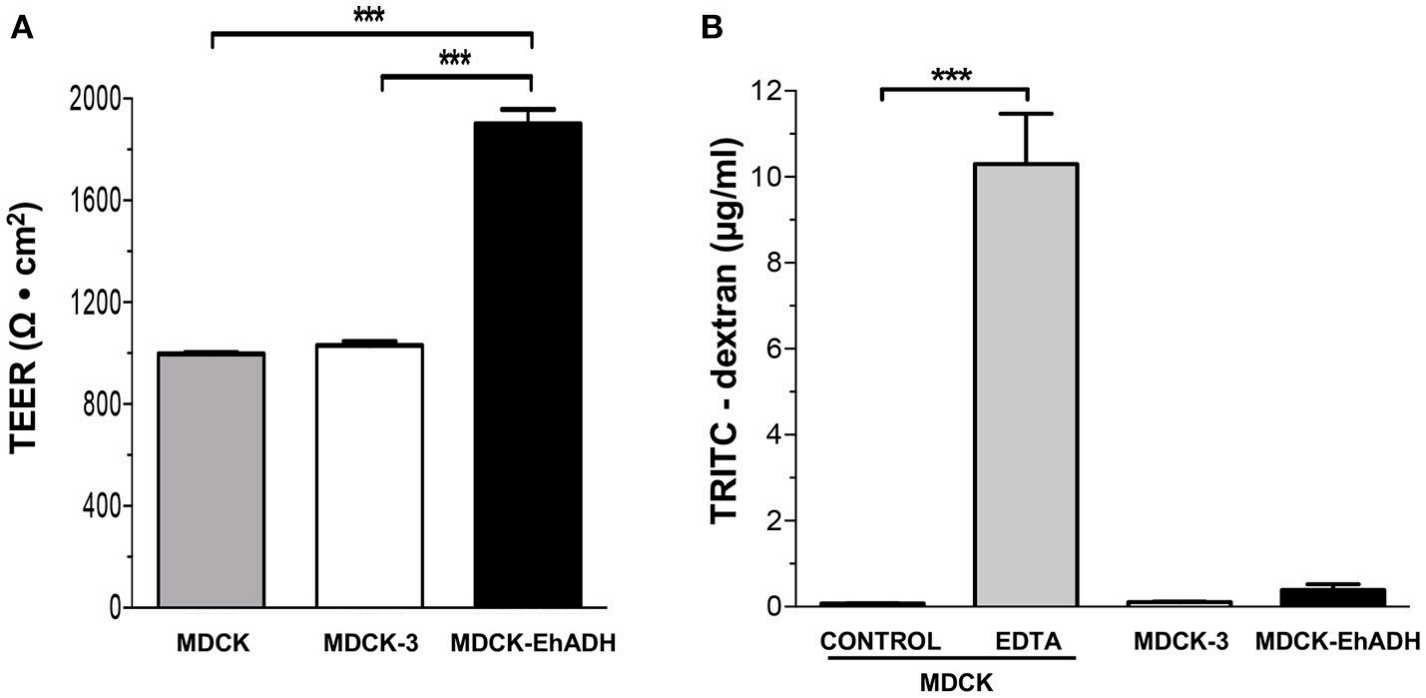
Permeability of MDCK-EhADH cells. **(A)** Non-transfected and transfected MDCK cells were seeded in Transwell filters and when they reached confluence, TEER was monitored. Background resistance from empty filters was deducted for each value. **(B)** Non-transfected and transfected MDCK cells were seeded in Transwell filters and when they reached confluence, the paracellular flux of 4 kDa TRITC-dextran was measured from the apical to the basolateral direction. MDCK cells treated with 5 mM EDTA were used as positive control. Data represent the mean and standard error from three independent experiments by triplicate. ^***^p < 0.001, according to two-tailed Student *t*-test.

Previously, we reported that EhCPADH and EhCP112 affect the gate function of TJ in epithelial cells (Cuellar et al., 2017; Hernández-Nava et al., 2017). Therefore, we wondered if EhADH expression on MDCK cells may alter this function, characterized by the regulation of ion and macromolecules flux. To evaluate this, we used transfected epithelial cells cultured in Transwell filters. MDCK-EHADH cells exhibited approximately twice higher TEER values compared to MDCK-3 cells (Figure 8A). To measure the macromolecules flux, the non-ionic dextran marker coupled to the TRITC-fluorescent dye was added to the apical side of epithelial monolayers and then, we quantified the tracer diffusion across the paracellular pathway from the apical to the basolateral side. MDCK-EhADH and MDCK-3 cells presented a low dextran permeability, similar to non-transfected MDCK cells (Figure 8B). As a positive control, cells were incubated with EDTA that disassembles epithelial junctions (Deli, 2009). MDCK cells treated with EDTA allowed the free passage of dextran between intercellular spaces. Our data showed that EhADH enhanced the electrical tightness of MDCK monolayers, but it had not impact on macromolecules permeability.

### EhADH co-localizes and increases the amount of TJ proteins in MDCK-EhADH cells

**Figure 9:**
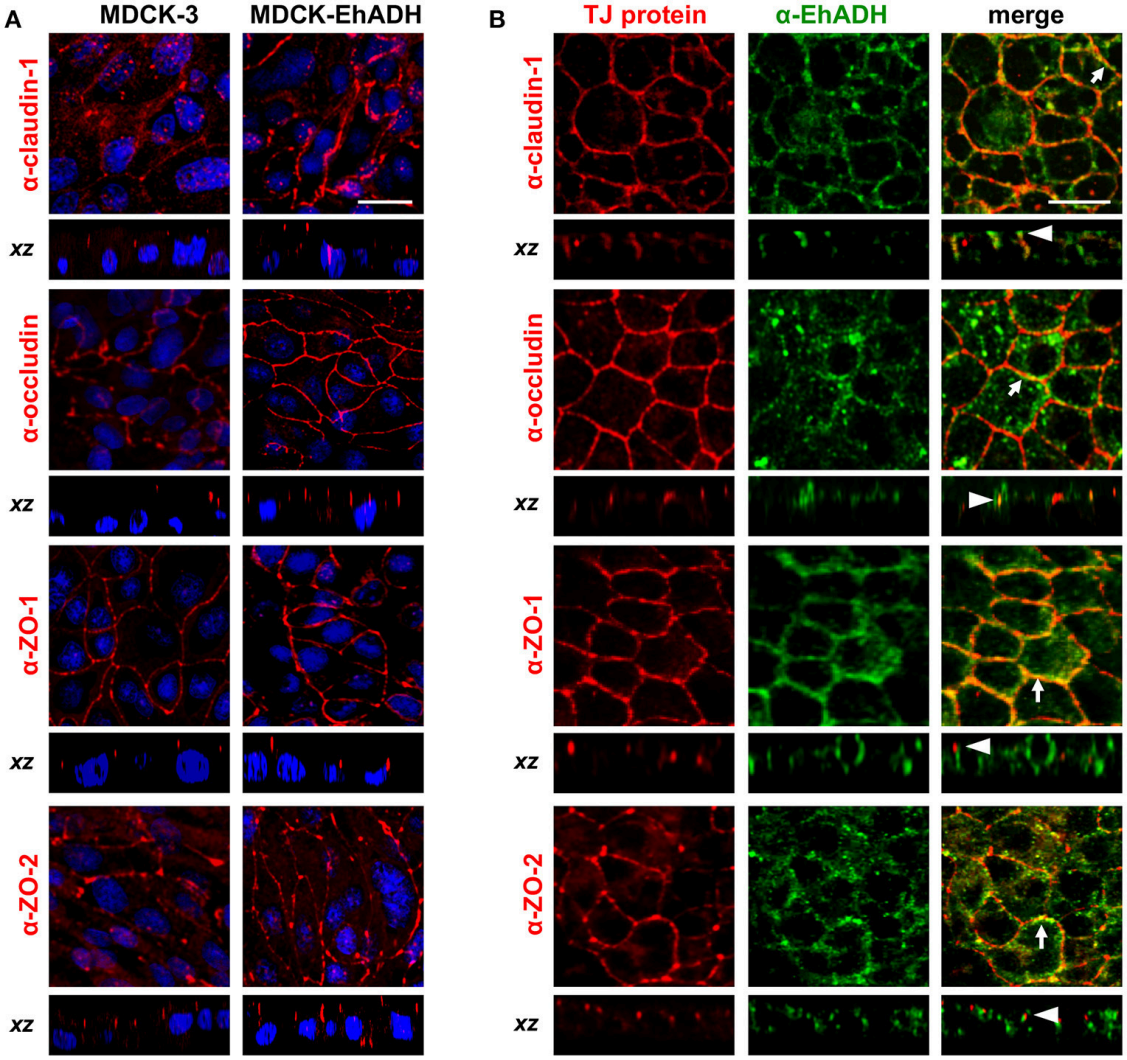
Effect of EhADH expression on TJ proteins localization. **(A)** Confluent transfected MDCK cells were fixed, permeabilized and stained with α-claudin-1, α-occludin, α-ZO-1 or α-ZO-2 antibodies, followed by the corresponding TRITC-secondary antibodies. Nuclei were counterstained with DAPI (blue). **(B)** MDCK-EhADH cells were stained with α-claudin-1, α-occludin, α-ZO-1 or α-ZO-2 (red) and α-EhADH (green) antibodies, followed by the corresponding TRITC- and FITC-secondary antibodies, respectively. Arrows: co-localization at the plasma membrane. Arrowheads: co-localization at the TJ region. Representative images of *xy*- and *xz*-optical planes observed by confocal microscopy. Bar = 20 μm.

The above results evidenced that MDCK-EhADH monolayers have a higher TEER than MDCK-3 cells. TJ functions depend on the localization and amount of proteins in the intercellular space. Thus, to analyse the effect of EhADH expression in the localization of junctional proteins, we performed immunofluorescence assays using α-claudin-1, α-occludin, α-ZO-1, α- ZO-2, and α-EhADH antibodies. In contrast to the results obtained using the rEhADH added to the apical side of cell monolayers (Figures 2, 3), confocal images showed that EhADH expression in MDCK cells did not affect the localization of proteins at TJ region, as it was clearly evidenced at *xz*-planes (Figure 9A). However, MDCK-EhADH cells presented an increase of the fluorescence corresponding to claudin-1, occludin, ZO-1 and ZO-2 (Figure 9A). As expected, all TJ proteins studied here co-localized with EhADH at the TJ region (Figure 9B). These results suggested that the stable presence of EhADH in transfected cells provoked an increase of claudin-1, occludin, ZO-1, and ZO-2 at TJ.

**Figure 10:**
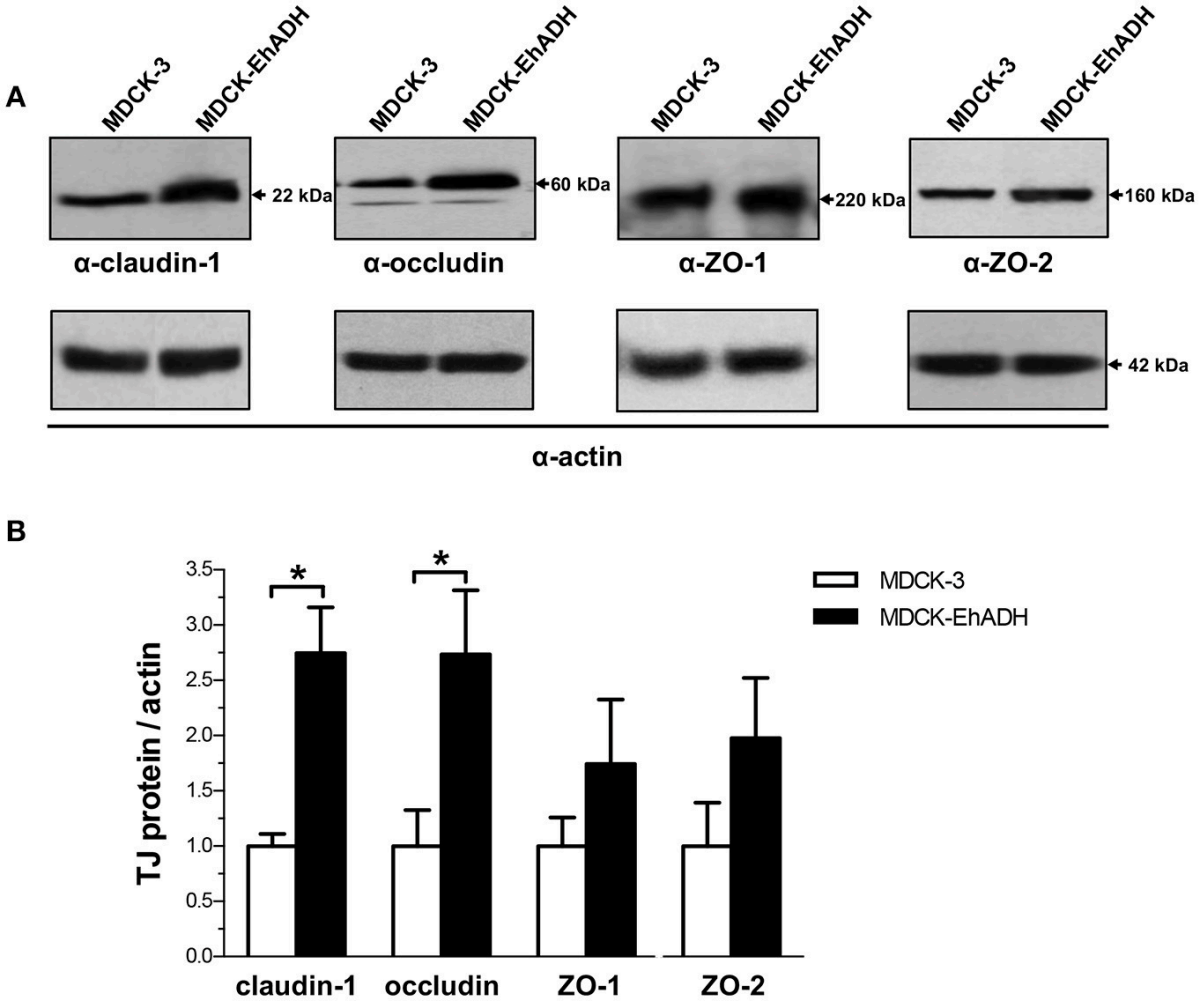
Effect of EhADH expression on the amount of TJ proteins. **(A)** Total extracts of confluent transfected MDCK cells were separated on SDS-PAGE, transferred to nitrocellulose membranes and blotted with α-claudin-1, α-occludin, α-ZO-1 or α-ZO-2 antibodies (upper panels). Lower panels: as loading controls, same membranes were stripped and blotted with the α-actin antibody (representative images). **(B)** The specific amount of TJ proteins was obtained as the ratio of TJ proteins between actin densitometric evaluations from **(A)**. Data represent the mean values and standard error from three independent experiments. ^*^p < 0.05, according to two-tailed Student *t*-test.

To further confirm the increased amount of TJ proteins in transfected cells, we analyzed their expression by western blot assays using the corresponding antibodies. Quantification of western blot results revealed that the amount of claudin-1 and occludin increased around 2.5 times in MDCK-EhADH cells, in comparison to MDCK-3 cells (Figure 10). Meanwhile, ZO-1 and ZO-2 showed a slight increase in MDCK-EhADH cells without significant differences when compared to MDCK-3 cells. Actin was used as loading control and all densitometric analysis were normalized regarding this protein. These results correlated with the immunofluorescence findings, confirming that EhADH expression provoked a significant increase of claudin-1 and occludin, which are mainly responsible for the TJ gate function.

### EhADH expression in MDCK cells facilitates epithelial destruction by trophozoites

**Figure 11:**
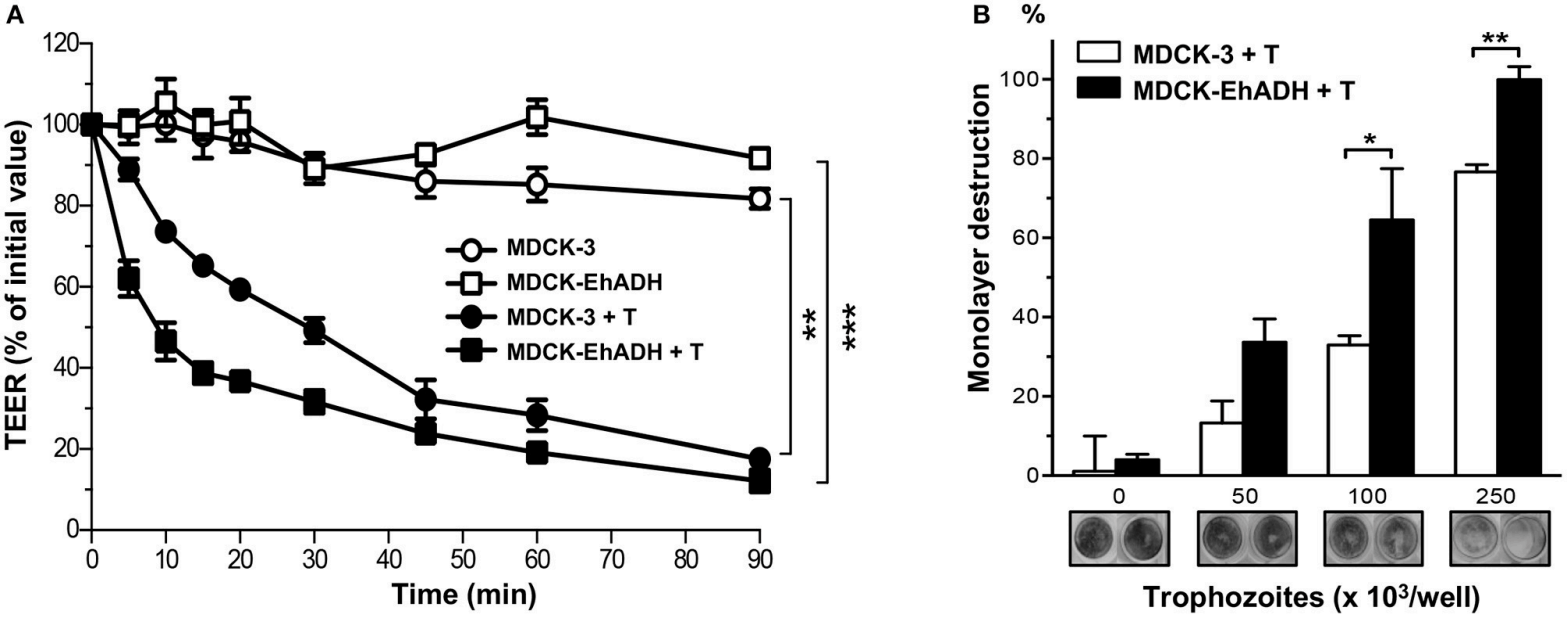
Effect of trophozoites on MDCK cells expressing EhADH. **(A)** Transfected MDCK cells were seeded in Transwell filters and when they reached confluence, trophozoites (10^5^/cm^2^) were apically added. TEER was monitored for 2 h and data were normalized according to the initial value given by each Transwell. Means and standard errors represent each time point from three independent assays performed by triplicate. ^**^p < 0.01 and ^***^p < 0.001, according to two-tailed Student *t*-test. **(B)** Transfected confluent MDCK cells were incubated with 0, 50, 100, and 250 × 10^3^ live trophozoites. Monolayers cell destruction was determined after 2 h incubation by methylene blue staining. Then, dye was eluted and quantified by spectrophotometry. Data represent the mean and standard error of three independent experiments by triplicate statistically analyzed by two-tailed Student *t*-test. ^*^p < 0.05, ^**^p < 0.01. Representative images of monolayers destruction are showed below the graph.

One of the first signs of damage to MDCK cell monolayers during *E. histolytica* invasion is TEER dropping. After this, cells are detached from the substrate and destroyed by the parasite (Martínez-Palomo et al., 1985). To evaluate the effect of *E. histolytica* trophozoites over transfected MDCK cells, epithelial cells were grown in Transwell filters until confluence, and live trophozoites were added in the apical side of monolayers, then, TEER was monitored during 90 min. To compare the TEER behavior of transfected cells, we normalized each TEER value regarding the one registered at 0 min. Results showed a stable TEER performance by MDCK-EhADH and MDCK-3 cells non-incubated with parasites (Figure 11A). Meanwhile, a TEER drop was evident when transfected cells were incubated with trophozoites. However, TEER values were lower in MDCK-EhADH than in MDCK-3 cells, from 5 to 30 min incubation. After this time, the effect of trophozoites on TEER seemed indistinct between MDCK-3 and MDCK-EhADH cells, reaching a stable behavior from 45 to 90 min.

To visualize and determine epithelial destruction by live trophozoites on transfected MDCK cells, confluent monolayers were apically incubated with different number of parasites. Live trophozoites destroyed 33, 65, and 100% (using 50, 100, and 250 × 10^3^ amoebas, respectively) more MDCK-EhADH than MDCK-3 cells monolayers (Figure 11B). Representatives images of methylene blue-stained monolayers evidenced the damage produced by parasites. Surprisingly, trophozoites displayed more damage on MDCK cells expressing EhADH than MDCK-3 cells, suggesting that this protein facilitated the epithelial injury.

In summary, all results of this work suggested that EhADH altered TJ of the host epithelium, reaching the paracellular space, being internalized mainly by clathrin-coated vesicles, delocalizing and increasing TJ proteins, for eventually making epithelial cells more susceptible to other trophozoite effector proteins. Nevertheless, this hypothesis should be further addressed to get in depth on EhADH action mechanisms in this event.

## Discussion

*E. histolytica* trophozoites use several molecules to invade the host epithelium. Among them, our group has been investigating the participation of the EhCPADH complex in the breaking of intercellular junctions. We have found that EhCPADH and also EhCP112 reach the paracellular pathway by opening intercellular junctions and eventually, both proteins penetrate epithelial cells. The damage begins by affecting proteins and functions of TJ, and then, it continues degrading AJ and DSM proteins (Betanzos et al., 2013; Cuellar et al., 2017; Hernández-Nava et al., 2017). Nevertheless, the EhADH contribution to epithelium impairment during trophozoite invasion, had not been yet elucidated. In this work, by using a recombinant protein we revealed that EhADH gets first into the intercellular space, and next, it enters into the epithelial cells mainly by clathrin-coated vesicles. To analyse the effect of EhADH inside host cells, we generated MDCK cells stably expressing this adhesin. The EhADH expression produced more adhesiveness to target cells and an enhanced TEER due to a higher amount of claudin-1 and occludin. Interestingly, the EhADH expression in MDCK cells facilitated the epithelial damage produced by live trophozoites, suggesting a putative role for EhADH in synergizing the effect of other *E. histolytica* molecules during host invasion.

EhCPADH and its components are secreted to the medium (García-Rivera et al., 1999; Ocádiz et al., 2005; Bolaños et al., 2016), and together with other parasite molecules (Lejeune et al., 2011; Cornick et al., 2016) constitute an efficient mechanism to reach and effectively damage the host epithelium. We previously demonstrated that the EhCPADH complex drops TEER and interacts with claudin-1 and occludin, to eventually degrade them along with ZO-1 and ZO-2 (Betanzos et al., 2013). Besides, we evaluated the participation of EhCP112, the proteolytic part of this complex, by producing a recombinant enzyme (rEhCP112) which reaches the apical side of epithelium and then invade it through intercellular junctions (Cuellar et al., 2017; Hernández-Nava et al., 2017). Here, we investigated if the other part of the EhCPADH complex, the EhADH adhesin, also extended into the paracellular pathway, by using a recombinant protein (rEhADH, Figure 1) and characterizing its effect on epithelial cells. When rEhADH was added to the apical side of MDCK cells, it was at cellular borders co-localizing with occludin and claudin-1 at TJ, and eventually penetrated the cell (Figures 2, 3). These findings were consistent to that observed in Caco-2 cells (Figure 4), which have been used in other works for resembling the *E. histolytica* natural colonization site in host (Li et al., 1994; Ralston et al., 2014). Here, we preferred the MDCK cells model because this cell line is a friendly and easy system to study molecules and functions of TJ, and also to stably and efficiently transfect heterologous genes (Cereijido et al., 1978; Furuse et al., 2001; Dukes et al., 2011). In addition, they have been widely used as a model to characterize trophozoites damage and TEER dropping during epithelial invasion (Martínez-Palomo et al., 1985; Dolabella et al., 2012; Betanzos et al., 2013; Hernández-Nava et al., 2017).

To investigate the EhADH internalization mechanism, we followed this adhesin together with clathrin and caveolin-1, resulting in a higher co-localization with clathrin than with caveolin-1 (Figure 5A). The rEhADH endocytosis mediated by clathrin-coated vesicles was confirmed by using sucrose as an inhibitor (Mosso et al., 2008), which diminished these vesicles and rEhADH internalization, thus preventing occludin delocalization from the membrane (Figure 5B). The endocytosis mechanism mediated by clathrin-coated vesicles has been observed for other pathogens adhesins, such as AfaD of diarrhea-associated *E. coli* strains or HadA of *Haemophilus influenzae* (Jouve et al., 1997; Serruto et al., 2009). Moreover, similarly to EhADH, EhCP112 is transported by clathrin-coated vesicles, although this enzyme is also internalized by caveolae (Hernández-Nava et al., 2017).

In order to analyse the role of EhADH inside epithelial cells and also to eliminate the noise coming from other amoebic proteins that produce host cell damage, we performed the heterologous expression of this adhesin by transfecting MDCK cells with the *pcDNA3-ehadh* construct. MDCK-EhADH cells efficiently expressed the *ehadh* transcript and the corresponding protein with the expected 75 kDa molecular weight, maintaining a similar growing rate than control cells (Figures 6A–C). As well as in trophozoites (García-Rivera et al., 1999; Avalos-Padilla et al., 2015), EhADH was localized in cytoplasmic vesicles and at the plasma membrane of epithelial cells (Figure 6D).

EhADH possesses adhesive properties, facilitating trophozoites adhesion to target cells (García-Rivera et al., 1999; Bañuelos et al., 2012). Here, we demonstrated that MDCK-EhADH cells aggregated more among them, and adhered more erythrocytes than control cells (Figure 7). The observed adhesive features were specific, since the mAbAdh antibody directed against EhADH inhibited them (Figure 7). Our findings suggested that MDCK-EhADH cells acquired the adhesive properties displayed by this trophozoite protein. A similar effect was also reported for the *H. influenzae* HadA adhesin during its heterologous expression in a non-invasive *E. coli* strain, which gained properties for aggregation and adherence to human epithelial cells and to extracellular matrix proteins (collagens I and III, fibronectin and laminin) (Serruto et al., 2009). On the other hand, it was not surprising that MDCK cells adhered erythrocytes, since epithelial cells are similar to erythroid cells and both contain similar membrane-cytoskeletal components (Bennett and Lorenzo, 2013). In addition, both cell types contain ankyrin and fodrin, involved in lateral membrane association upon cell-cell contact induction (Glenney and Glenney, 1983; Bennett and Lorenzo, 2013).

The increased adhesiveness among MDCK-EhADH cells and the presence of EhADH at cellular borders, affected the TJ gate function evidenced as an augment of TEER (Figure 8). In agreement, macromolecules flux by the paracellular pathway remained low. Other amoeba as *Acanthamoeba* also produced a high TEER in MDCK cells, probably because claudin-4 was increased and re-targeted to TJ, while claudin-2 diminished and was removed from the cellular borders (Flores-Maldonado et al., 2017). TEER is a measurement of ions flux through the paracellular pathway, and this flux is mainly regulated by claudins in different fashions; for example, claudin-4 promotes a barrier function by forming an ion channel, whereas claudin-2, constitutively expressed in the most leaky-epithelia, makes paracellular cation and water channels (Barmeyer et al., 2017). Therefore, we evaluated the localization and expression of TJ integral (claudin-1 and occludin) and scaffold (ZO-1 and ZO-2) proteins. According to TEER results, by immunofluorescence and western blot assays in MDCK-EhADH cells we observed an increase of the analyszd proteins at cellular borders, co-localizing with EhADH at the TJ region (Figure 9), although the augment was only significant for claudin-1 and occludin (Figure 10), molecules responsible for the gate function (Liang and Weber, 2014). Thereby, the delicate nature of the molecular composition of junctions and their balance, could be influencing the epithelium susceptibility for alterations due to pathogens and their molecules.

The effect produced by EhADH inside epithelial cells could be explained by its scaffold properties, characteristic of ALIX family protein members (Bissig and Gruenberg, 2014). TJ peripheral scaffold proteins link transmembrane and functional barrier proteins to the actomyosin-ring (Liang and Weber, 2014). In fact, in immortalized rat choroid plexus cells, Alix protein guarantees the proper assembly and actomyosin-ring positioning at the TJ region by interacting with F-actin, Par-3 and ZO-1, thus contributing to the maintenance of epithelial cell polarity and barrier function (Campos et al., 2016). Otherwise, from previous experiments, we demonstrated that an EhADH recombinant protein including the adherence epitope, interacts with a 97 kDa membrane protein of MDCK cells (Martinez-Lopez et al., 2004). We hypothesized this protein could correspond to a claudin-1 tetramer, since claudins are capable to form oligomers, which are responsible for junction selectivity (Coyne et al., 2003). Furthermore, EhCPADH binds to claudin-1, occludin, ZO-1 and ZO-2 in MDCK cells (Betanzos et al., 2013). Thus, our findings in this work suggested that EhADH could be acting as a peripheral scaffolding protein to reinforce the attachment of claudin-1 and occludin to the TJ region, probably tightening cell-cell contacts. This assumption can be reinforced by the fact that other amoeba proteins also act as intercellular junction molecules. *Dictyostelium discoideum* amoeba contains an adhesion molecule named Aardvark, which is similar to the AJ scaffold protein β-catenin, suggesting the presence of a rudimentary cell-cell adhesion during the formation of the fruiting body that develops in colonies deprived of nutrients (Grimson et al., 2000). Even more, *E. histolytica* expresses an occludin-like protein that can alter the colonic epithelial barrier (Goplen et al., 2013).

Interestingly, MDCK-EhADH cells incubated with live trophozoites were more susceptible to parasites damage, according to TEER and cytopathic experiments (Figure 11). Although, these cells developed higher TEER values than control cells, trophozoites dropped TEER in MDCK-EhADH cells more than in MDCK-3 cells (from 5 to 30 min amoeba incubation); in accordance, parasites caused more damage on monolayers expressing EhADH. In trophozoites, it has been reported that EhADH binds to diverse molecules such as EhCP112, EhVps32, EhNPC1, EhNPC2, LBPA and cholesterol (Avalos-Padilla et al., 2015; Bolaños et al., 2016; Castellanos-Castro et al., 2016; Cuellar et al., 2017), thus participating in distinct virulence events. Therefore, in MDCK-EhADH cells, we hypothesized that this adhesin could be modulating or associating to these molecules or others, to facilitate their participation on epithelial damage. Identity of these putative molecules and related mechanisms should be further investigated.

Summarizing our findings from previous reports and those obtained in this paper, we demonstrated that EhCPADH and its components by separate, could reach the intercellular space and affect the epithelial barrier function. In the case of EhADH, when it is secreted by trophozoites to the medium, extends toward the paracellular pathway and later, after its internalization, modulates the expression and localization of claudin-1 and occludin. Once inside, EhADH could prepare host cells for the action of other virulence factors, making the epithelium more susceptible to the trophozoite attack.

## Author contributions

AB and EO: Designed, performed and analyzed experiments, and wrote the manuscript. DZ: Carried out immunofluorescence assays. EH-N: Produced the recombinant protein. PC: Performed RT-PCR experiments. CB: Contributed to experiments analysis, discussion and manuscript writing.

### Conflict of interest statement

The authors declare that the research was conducted in the absence of any commercial or financial relationships that could be construed as a potential conflict of interest.

## Supplementary material

Figure S1 Co-localization of rEhADH with claudin-1 on sparse MDCK cells. rEhADH coupled to Alexa 647 (green) was apically added to sparse MDCK cells and incubated for different times, then, cells were fixed and processed for immunofluorescence assays using the α-claudin-1 antibody (red). Nuclei were counterstained with DAPI (blue) and preparations were analyzed through a confocal microscope at *xy-* and *zy-*planes. Arrows: co-localization at cellular borders. Full arrowheads: co-localization at lateral membrane. Empty arrowheads: localization of EhADH (green) and claudin-1 (red) at cytoplasm. Bar = 20 μm.

Figure S2 rEhADH localization in MDCK cells where clathrin-transport was inhibited. MDCK cells were incubated for 1 h with DMEM medium (–) or 300 mM sucrose (+) at 37°C and then rEhADH coupled to Alexa 647 (green) was apically added for 1 min. Preparations were analyzed through a confocal microscope at *xy-* and *xz-*planes. Arrows: rEhADH localization. Bar = 20 μm.

## References

[B1] Arroyo R. Orozco E. (1987). Localization and identification of an *Entamoeba histolytica* adhesin. Mol. Biochem. Parasitol. 23, 151–158. 10.1016/0166-6851(87)90150-2 2883572

[B2] Avalos-Padilla Y. Betanzos A. Javier-Reyna R. Garcia-Rivera G. Chávez-Munguía B. Lagunes-Guillen A.. (2015). EhVps32 is a vacuole-associated protein involved in pinocytosis and phagocytosis of *Entamoeaba histolytica*. PLoS Pathog. 11:e1005079. 10.1371/journal.ppat.1005079 26230715 PMC4521941

[B3] Avalos-Padilla Y. Knorr R. L. Javier-Reyna R. García-Rivera G. Lipowsky R. Dimova R.. (2018). The Conserved ESCRT-III machinery participates in the phagocytosis of *Entamoeba histolytica*. Front. Cell. Infect. Microbiol. 8:53. 10.3389/fcimb.2018.00053 29546036 PMC5838018

[B4] Azuara-Liceaga E. Betanzos A. Cardona-Felix C. S. Castañeda-Ortiz E. J. Cárdenas H. Cárdenas-Guerra R. E.. (2018). The Sole DNA ligase in *Entamoeba histolytica* is a high-fidelity DNA ligase involved in DNA damage repair. Front. Cell. Infect. Microbiol. 8:214. 10.3389/fcimb.2018.00214 30050869 PMC6052137

[B5] Bañuelos C. García-Rivera G. López-Reyes I. Mendoza L. González-Robles A. Herranz S.. (2012). EhADH112 Is a Bro1 domain-containing protein involved in the *Entamoeba histolytica* multivesicular bodies pathway. J. Biomed. Biotechnol. 2012:657942. 10.1155/2012/657942. 22500103 PMC3303925

[B6] Bañuelos C. García-Rivera G. López-Reyes I. Orozco E. (2005). Functional characterization of EhADH112: an *Entamoeba histolytica* Bro1 domain-containing protein. Exp Parasitol. 110, 292–297. 10.1016/j.exppara.2005.03.005 15955327

[B7] Barmeyer C. Fromm M. Schulzke J. D. (2017). Active and passive involvement of claudins in the pathophysiology of intestinal inflammatory diseases. Pflugers Arch. Eur. J. Physiol. 469, 15–26. 10.1007/s00424-016-1914-6 27904960

[B8] Bennett V. Lorenzo D. N. (2013). Spectrin- and ankyrin-based membrane domains and the evolution of vertebrates. Curr. Top. Membr. 72, 1–37. 10.1016/B978-0-12-417027-8.00001-5 24210426

[B9] Betanzos A. Javier-Reyna R. García-Rivera G. Bañuelos C. González-Mariscal L. Schnoor M.. (2013). The EhCPADH112 Complex of *Entamoeba histolytica* interacts with tight Junction proteins occludin and claudin-1 to produce epithelial damage. PLoS ONE 8:e065100. 10.1371/journal.pone.0065100 23762290 PMC3676397

[B10] Bissig C. Gruenberg J. (2014). ALIX and the multivesicular endosome: ALIX in Wonderland. Trends Cell Biol. 24, 19–25. 10.1016/j.tcb.2013.10.009 24287454

[B11] Bolaños J. Betanzos A. Javier-Reyna R. García- Rivera G. Huerta M. Pais-Morales J.. (2016). EhNPC1 and EhNPC2 proteins participate in trafficking of exogenous cholesterol in *Entamoeba histolytica* trophozoites: relevance for phagocytosis. PLoS Pathog. 12:e1006089. 10.1371/journal.ppat.1006089 28002502 PMC5176366

[B12] Bracha R. Mirelman D. (1984). Virulence of *Entamoeba histolytica* trophozoites. Effects of bacteria, microaerobic conditions, and metronidazole. J. Exp. Med. 160, 353–368. 10.1084/jem.160.2.353 6088660 PMC2187461

[B13] Campos Y. Qiu X. Gomero E. Wakefield R. Horner L. Brutkowski W.. (2016). Alix-mediated assembly of the actomyosin-tight junction polarity complex preserves epithelial polarity and epithelial barrier. Nat. Commun. 7:11876. 10.1038/ncomms11876 27336173 PMC4931029

[B14] Capaldo C. T. Farkas A. E. Nusrat A. (2014). Epithelial adhesive junctions. F1000Prime Rep. 6:1. 10.12703/P6-1 24592313 PMC3883420

[B15] Cardona-Felix C. S. Lara-Gonzalez S. Brieba L. G. (2011). Structure and biochemical characterization of proliferating cellular nuclear antigen from a parasitic protozoon. Acta Crystallogr. D Biol. Crystallogr. 67, 497–505. 10.1107/S0907444911010547 21636889

[B16] Castellanos-Castro S. Cerda-García-Rojas C. M. Javier-Reyna R. Pais-Morales J. Chávez-Munguía B. Orozco E. (2016). Identification of the phospholipid lysobisphosphatidic acid in the protozoan *Entamoeba histolytica*: an active molecule in endocytosis. Biochem. Biophys. Rep. 5, 224–236. 10.1016/j.bbrep.2015.12.010 28955828 PMC5600446

[B17] Cereijido M. Robbins E. S. Dolan W. J. Rotunno C. A. Sabatini D. D. (1978). Polarized monolayers formed by epithelial cells on a permeable and translucent support. J. Cell Biol. 77, 853–880. 10.1083/jcb.77.3.853 567227 PMC2110138

[B18] Chadee K. Petri W. A Jr. Innes D. J. Ravdin J. I. (1987). Rat and human colonic mucins bind to and inhibit adherence lectin of *Entamoeba histolytica*. J. Clin. Invest. 80, 1245–1254. 10.1172/JCI113199 2890655 PMC442377

[B19] Cornick S. Chadee K. (2017). *Entamoeba histolytica*: Host parasite interactions at the colonic epithelium. Tissue Barriers 5:e1283386. 10.1080/21688370.2017.1283386 28452682 PMC5362996

[B20] Cornick S. Moreau F. Chadee K. (2016). *Entamoeba histolytica* cysteine proteinase 5 evokes mucin exocytosis from colonic goblet cells via αvβ3 integrin. PLoS Pathog. 12:e1005579. 10.1371/journal.ppat.1005579 27073869 PMC4830554

[B21] Coyne C. B. Gambling T. M. Boucher R. C. Carson J. L. Johnson L. G. (2003). Role of claudin interactions in airway tight junctional permeability. Am. J. Physiol. Lung Cell. Mol. Physiol. 285, L1166–L1178. 10.1152/ajplung.00182.2003 12909588

[B22] Cuellar P. Hernández-Nava E. García-Rivera G. Chávez-Munguía B. Schnoor M. Betanzos A.. (2017). *Entamoeba histolytica* EhCP112 dislocates and degrades claudin-1 and claudin-2 at tight junctions of the intestinal epithelium. Front. Cell. Infect. Microbiol. 7:372. 10.3389/fcimb.2017.00372 28861400 PMC5561765

[B23] Deli M. A. (2009). Potential use of tight junction modulators to reversibly open membranous barriers and improve drug delivery. Biochim. Biophys. Acta - Biomembr. 1788, 892–910. 10.1016/j.bbamem.2008.09.016 18983815

[B24] Diamond L. S. Harlow D. R. Cunnick C. C. (1978). A new medium for the axenic cultivation of *Entamoeba histolytica* and other Entamoeba. Trans. R Soc. Trop. Med. Hyg. 72, 431–432. 10.1016/0035-9203(78)90144-X 212851

[B25] Dolabella S. S. Serrano-Luna J. Navarro-García F. Cerritos R. Ximénez C. Galván-Moroyoqui J. M.. (2012). Amoebic liver abscess production by entamoeba dispar. Ann. Hepatol. 11, 107–117. 22166569

[B26] Dukes J. D. Whitley P. Chalmers A. D. (2011). The MDCK variety pack: Choosing the right strain. BMC Cell Biol. 12:43. 10.1186/1471-2121-12-43 21982418 PMC3209442

[B27] Flores-Maldonado C. González-Robles A. Salazar-Villatoro L. Omaña-Molina M. Gallardo J. M. González-Lázaro M.. (2017). Acanthamoeba (T4) trophozoites cross the MDCK epithelium without cell damage but increase paracellular permeability and transepithelial resistance by modifying tight junction composition. Exp. Parasitol. 183, 69–75. 10.1016/j.exppara.2017.10.013 29097064

[B28] Furuse M. Furuse K. Sasaki H. Tsukita S. (2001). Conversion of zonulae occludentes from tight to leaky strand type by introducing claudin-2 into madin-darby canine kidney I cells. J. Cell Biol. 153, 263–272. 10.1083/jcb.153.2.263 11309408 PMC2169456

[B29] García-Rivera G. Rodríguez M. A. Ocadiz R. Martinez-Lopez M. C. Arroyo R. Gonzalez-Robles A.. (1999). *Entamoeba histolytica* : a novel cysteine protease and an adhesin form the 112 kDa surface protein. Mol. Microbiol. 33, 556–568. 10.1046/j.1365-2958.1999.01500.x 10417646

[B30] Glenney J. R. Glenney P. (1983). Fodrin is the general spectrin-like protein found in most cells whereas spectrin and the TW protein have a restricted distribution. Cell 34, 503–512. 10.1016/0092-8674(83)90383-5 6352052

[B31] Goplen M. Lejeune M. Cornick S. Moreau F. Chadee K. (2013). *Entamoeba histolytica* contains an occludin-like protein that can alter colonic epithelial barrier function. PLoS ONE 8:e73339. 10.1371/journal.pone.0073339 24058468 PMC3772840

[B32] Grimson M. J. Coates J. C. Reynolds J. P. Shipman M. Blanton R. L. Harwood A. J. (2000). Adherens junctions and beta-catenin-mediated cell signalling in a non-metazoan organism. Nature 408, 727–731. 10.1038/35047099 11130075

[B33] Hernández-Nava E. Cuellar P. Nava P. Chávez-Munguía B. Schnoor M. Orozco E.. (2017). Adherens junctions and desmosomes are damaged by *Entamoeba histolytica*: participation of EhCPADH complex and EhCP112 protease. Cell Microbiol. 19:e12761. 10.1111/cmi.12761 28656597

[B34] Jouve M. Garcia M. I. Courcoux P. Labigne A. Gounon P. Le Bouguénec C. (1997). Adhesion to and invasion of HeLa cells by pathogenic Escherichia coli carrying the afa-3 gene cluster are mediated by the AfaE and AfaD proteins, respectively. Infect. Immun. 65, 4082–4089. 9317011 10.1128/iai.65.10.4082-4089.1997 PMC175587

[B35] Leippe M. (1997). Amoebapores. Parasitol. Today 13, 178–183. 10.1016/S0169-4758(97)01038-7 15275088

[B36] Lejeune M. Moreau F. Chadee K. (2011). Prostaglandin E2 produced by *Entamoeba histolytica* signals via EP4 receptor and alters claudin-4 to increase ion permeability of tight junctions. Am. J. Pathol. 179, 807–818. 10.1016/j.ajpath.2011.05.001 21683675 PMC3157226

[B37] Leroy A. Lauwaet T. De Bruyne G. Cornelissen M. Mareel M. (2000). *Entamoeba histolytica* disturbs the tight junction complex in human enteric T84 cell layers. FASEB J. 14, 1139–1146. 10.1096/fasebj.14.9.1139 10834936

[B38] Li E. Stenson W. F. Kunz-Jenkins C. Swanson P. E. Duncan R. Stanley S. L. Jr. (1994). *Entamoeba histolytica* interactions with polarized human intestinal Caco-2 epithelial cells. Infect. Immun. 62, 5112–5119. 7927794 10.1128/iai.62.11.5112-5119.1994 PMC303232

[B39] Liang G. H. Weber C. R. (2014). Molecular aspects of tight junction barrier function. Curr. Opin. Pharmacol. 19, 84–89. 10.1016/j.coph.2014.07.017 25128899 PMC4330960

[B40] Machado F. S. Rodriguez N. E. Adesse D. Garzoni L. R. Esper L. Lisanti M. P.. (2012). Recent developments in the interactions between caveolin and pathogens. Adv. Exp. Med. Biol. 729, 65–82. 10.1007/978-1-4614-1222-9_5 22411314 PMC3564053

[B41] Madriz X. Martínez M. B. Rodríguez M. A. Sierra G. Martínez-López C. Riverón A. M.. (2004). Expression in fibroblasts and in live animals of *Entamoeba histolytica* polypeptides EhCP112 and EhADH112. Microbiology 150, 1251–1260. 10.1099/mic.0.26938-0 15133088

[B42] Martinez-Lopez C. Orozco E. Sanchez T. Garcia-Perez R. M. Hernandez-Hernandez F. Rodriguez M. A. (2004). The EhADH112 recombinant polypeptide inhibits cell destruction and liver abscess formation by *Entamoeba histolytica* trophozoites. Cell Microbiol. 6, 367–376. 10.1111/j.1462-5822.2004.00363.x 15009028

[B43] Martínez-Palomo A. Gonzalez-Robles A. Chávez B. Orozco E. Fernandez-Castelo S. Cervantes A. (1985). Structural bases of the cytolytic mechanisms of *Entamoeba histolytica*. J. Protozool. 32, 166–175. 10.1111/j.1550-7408.1985.tb03033.x 2859368

[B44] Meléndez-López S. G. Herdman S. Hirata K. Choi M. H. Choe Y. Craik C.. (2007). Use of recombinant *Entamoeba histolytica* cysteine proteinase 1 to identify a potent inhibitor of amebic invasion in a human colonic model. Eukaryot. Cell 6, 1130–1136. 10.1128/EC.00094-07 17513563 PMC1951106

[B45] Montaño S. Orozco E. Correa-Basurto J. Bello M. Chávez-Munguía B. Betanzos A. (2017). Heterodimerization of the *Entamoeba histolytica* EhCPADH virulence complex through molecular dynamics and protein–protein docking. J. Biomol. Struct. Dyn. 35, 486–503. 10.1080/07391102.2016.1151831 26861050

[B46] Moreno-Ruiz E. Galán-Díez M. Zhu W. Fernández-Ruiz E. D'Enfert C. Filler S. G.. (2009). Candida albicans internalization by host cells is mediated by a clathrin-dependent mechanism. Cell Microbiol. 11, 1179–1189. 10.1111/j.1462-5822.2009.01319.x 19416270 PMC4098847

[B47] Morita E. Sandrin V. Chung H. Y. Morham S. G. Gygi S. P. Rodesch C. K.. (2007). Human ESCRT and ALIX proteins interact with proteins of the midbody and function in cytokinesis. EMBO J. 26, 4215–4227. 10.1038/sj.emboj.7601850 17853893 PMC2230844

[B48] Mosso C. Galván-Mendoza I. J. Ludert J. E. del Angel R. M. (2008). Endocytic pathway followed by dengue virus to infect the mosquito cell line C6/36 HT. Virology 378, 193–199. 10.1016/j.virol.2008.05.012 18571214

[B49] Ocádiz R. Orozco E. Carrillo E. Quintas L. I. Ortega-Lopez J. Garcia-Perez R. M.. (2005). EhCP112 is an *Entamoeba histolytica* secreted cysteine protease that may be involved in the parasite-virulence. Cell Microbiol. 7, 221–232. 10.1111/j.1462-5822.2004.00453.x 15659066

[B50] Odorizzi G. (2006). The multiple personalities of Alix. J. Cell Sci. 119, 3025–3032. 10.1242/jcs.03072 16868030

[B51] Orozco E. Guarneros G. Martinez-Palomo A. Sanchez T. (1983). *Entamoeba histolytica*. Phagocytosis as a virulence factor. J. Exp. Med. 158, 1511–1521. 10.1084/jem.158.5.1511 6313842 PMC2187140

[B52] Ralston K. S. Solga M. D. Mackey-Lawrence N. M. Somlata Bhattacharya A. Petri W. A. (2014). Trogocytosis by *Entamoeba histolytica* contributes to cell killing and tissue invasion. Nature 508, 526–530. 10.1038/nature13242 24717428 PMC4006097

[B53] Rosenberger C. M. Brumell J. H. Finlay B. B. (2000). Microbial pathogenesis: lipid rafts Rosenberger et al., 2000 as pathogen portals. Curr. Biol. 10, R823–R825. 10.1016/S0960-9822(00)00788-0 11102822

[B54] Sambuy Y. De Angelis I. Ranaldi G. (2005). The Caco-2 cell line as a model of the intestinal barrier: influence of cell and culture-related factors on Caco-2 cell functional characteristics. Cell Biol. 21, 1–26. 10.1007/s10565-005-0085-6 15868485

[B55] Serruto D. Spadafina T. Scarselli M. Bambini S. Comanducci M. Höhle S.. (2009). HadA is an atypical new multifunctional trimeric coiled-coil adhesin of Haemophilus influenzae biogroup aegyptius, which promotes entry into host cells. Cell Microbiol. 11, 1044–1063. 10.1111/j.1462-5822.2009.01306.x 19290916

[B56] Singh R. S. Walia A. K. Kanwar J. R. Kennedy J. F. (2016). Amoebiasis vaccine development: a snapshot on *E. histolytica* with emphasis on perspectives of Gal/GalNAc lectin. Int. J. Biol. Macromol. 91, 258–268. 10.1016/j.ijbiomac.2016.05.043 27181579

[B57] Thoreson M. A. Anastasiadis P. Z. Daniel J. M. Ireton R. C. Wheelock M. J. Johnson K. R.. (2000). Selective uncoupling of p120(ctn) from E-cadherin disrupts strong adhesion. J. Cell Biol. 148, 189–201. 10.1083/jcb.148.1.189 10629228 PMC2156209

