# Supplementary figures and images for: Epithelial Cells Expressing EhADH, An Entamoeba histolytica Adhesin, Exhibit Increased Tight Junction Proteins
Source: Front Cell Infect Microbiol. 2018 Sep 28;8:340. doi: 10.3389/fcimb.2018.00340 (PMC6172307; doi:10.3389/fcimb.2018.00340)

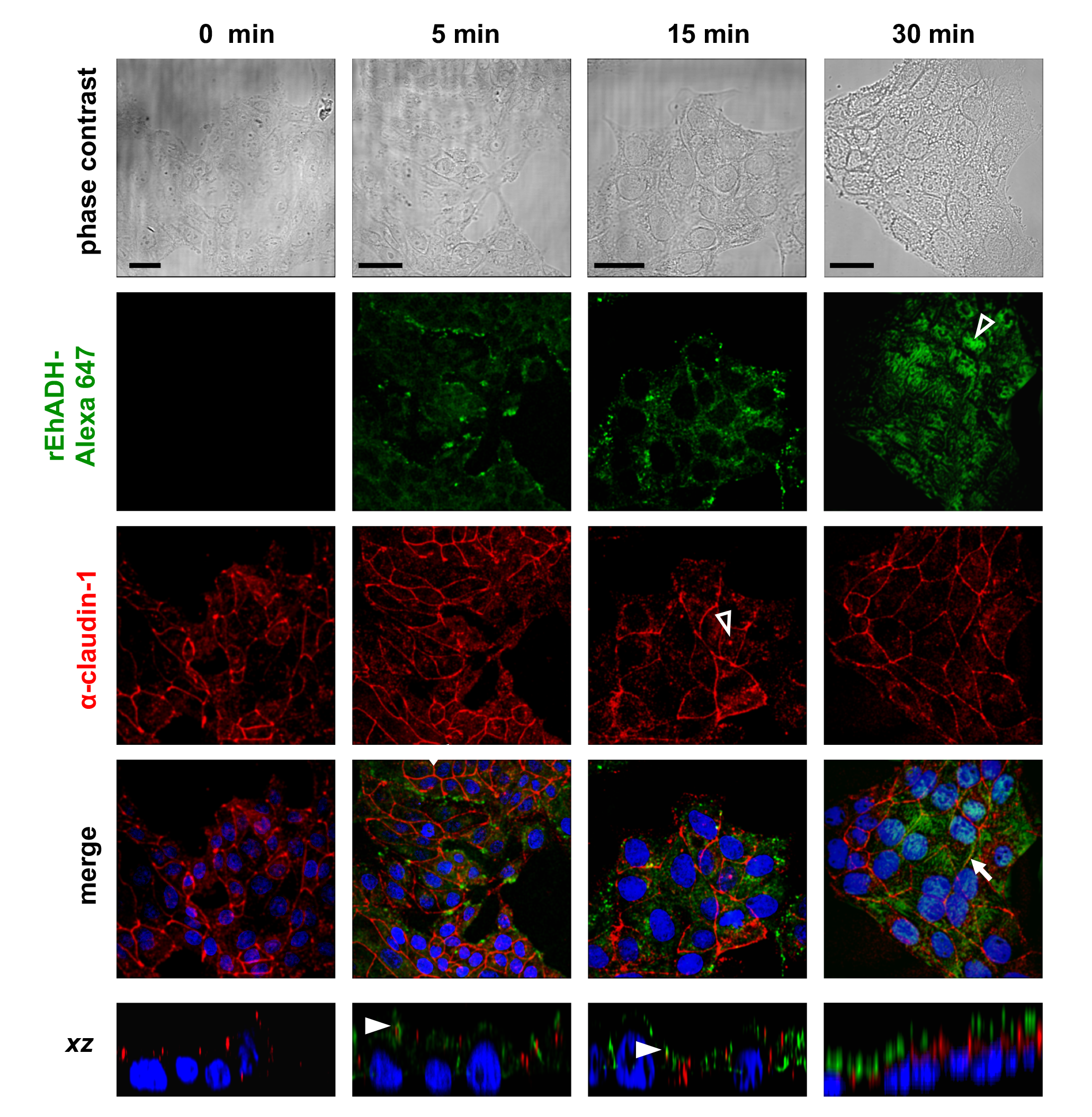

Supplement: Figure S1 — Co-localization of rEhADH with claudin-1 on sparse MDCK cells. rEhADH coupled to Alexa 647 (green) was apically added to sparse MDCK cells and incubated for different times, then, cells were fixed and processed for immunofluorescence assays using the α-claudin-1 antibody (red). Nuclei were counterstained with DAPI (blue) and preparations were analyzed through a confocal microscope at xy- and zy-planes. Arrows: co-localization at cellular borders. Full arrowheads: co-localization at lateral membrane. Empty arrowheads: localization of EhADH (green) and claudin-1 (red) at cytoplasm. Bar = 20 μm. [file Image_1.TIF]

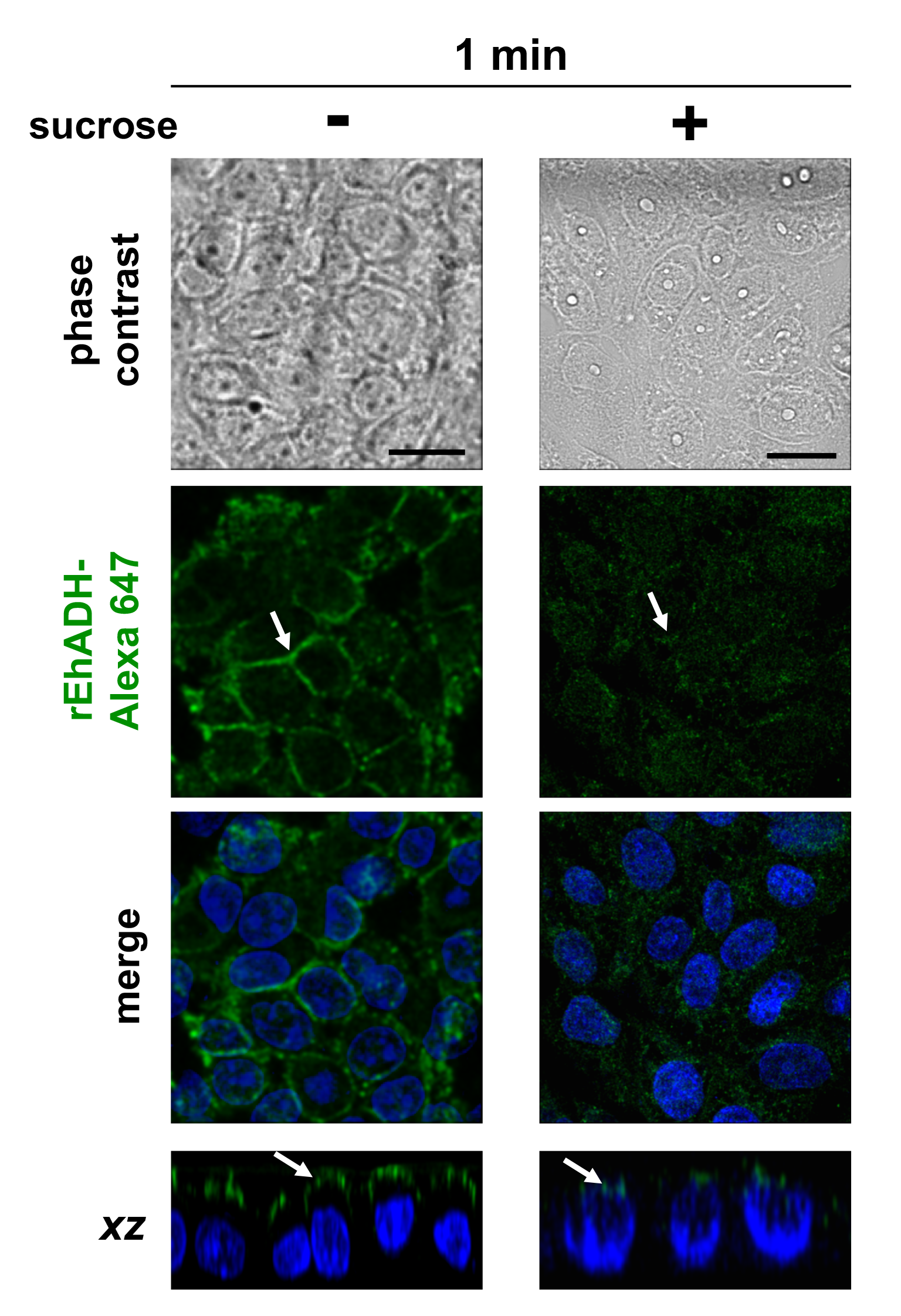

Supplement: Figure S2 — rEhADH localization in MDCK cells where clathrin-transport was inhibited. MDCK cells were incubated for 1 h with DMEM medium (–) or 300 mM sucrose (+) at 37°C and then rEhADH coupled to Alexa 647 (green) was apically added for 1 min. Preparations were analyzed through a confocal microscope at xy- and xz-planes. Arrows: rEhADH localization. Bar = 20 μm. [file Image_2.tif]
